# Genetic Diversity, Distribution, and Genomic Characterization of Antibiotic Resistance and Virulence of Clinical *Pseudomonas aeruginosa* Strains in Kenya

**DOI:** 10.3389/fmicb.2022.835403

**Published:** 2022-03-14

**Authors:** Shahiid Kiyaga, Cecilia Kyany'a, Angela W. Muraya, Hunter J. Smith, Emma G. Mills, Caleb Kibet, Gerald Mboowa, Lillian Musila

**Affiliations:** ^1^Department of Immunology and Molecular Biology, School of Biomedical Sciences, College of Health Sciences, Makerere University, Kampala, Uganda; ^2^Department of Emerging Infectious Diseases, United States Army Medical Research Directorate-Africa, Nairobi, Kenya; ^3^Center for Clinical Research, Kenya Medical Research Institute, Nairobi, Kenya; ^4^Department of Biochemistry, Jomo Kenyatta University of Agriculture and Technology, Nairobi, Kenya; ^5^Multidrug-Resistant Organism Repository and Surveillance Network (MRSN), Walter Reed Army Institute of Research, Silver Spring, MD, United States; ^6^Molecular Biology and Bioinformatics Unit, International Center for Insect Physiology and Ecology, Nairobi, Kenya; ^7^The African Center of Excellence in Bioinformatics and Data-Intensive Sciences, Infectious Diseases Institute, College of Health Sciences, Makerere University, Kampala, Uganda

**Keywords:** *Pseudomonas aeruginosa*, Kenya, sequence types, antimicrobial resistance, virulence

## Abstract

*Pseudomonas aeruginosa* is a leading cause of nosocomial infections worldwide. It can produce a range of debilitating infections, have a propensity for developing antimicrobial resistance, and present with a variety of potent virulence factors. This study investigated the sequence types (ST), phenotypic antimicrobial susceptibility profiles, and resistance and virulence genes among clinical isolates from urinary tract and skin and soft tissue infections. Fifty-six *P. aeruginosa* clinical isolates were obtained from six medical centers across five counties in Kenya between 2015 and 2020. Whole-genome sequencing (WGS) was performed to conduct genomic characterization, sequence typing, and phylogenetic analysis of the isolates. Results showed the presence of globally distributed high-risk clones (ST244 and ST357), local high-risk clones (ST2025, ST455, and ST233), and a novel multidrug-resistant (MDR) clone carrying virulence genes (ST3674). Furthermore, 31% of the study isolates were found to be MDR with phenotypic resistance to a variety of antibiotics, including piperacillin (79%), ticarcillin-clavulanic acid (57%), meropenem (34%), levofloxacin (70%), and cefepime (32%). Several resistance genes were identified, including carbapenemases *VIM-6* (ST1203) and *NDM-1* (ST357), fluoroquinolone genes, *crpP*, and *qnrVCi*, while 14 and 22 different chromosomal mutations were detected in the *gyrA* and *parC* genes, respectively. All isolates contained at least three virulence genes. Among the virulence genes identified, *phzB1* was the most abundant (50/56, 89%). About 21% (12/56) of the isolates had the *exoU+/exoS*- genotype, while 73% (41/56) of the isolates had the *exoS+/exoU*- genotype. This study also discovered 12 novel lineages of *P. aeruginosa*, of which one (ST3674) demonstrated both extensive antimicrobial resistance and the highest number of virulence genes (236/242, 98%). Although most high-risk clones were detected in Nairobi County, high-risk and clones of interest were found throughout the country, indicating the local spread of global epidemic clones and the emergence of new strains. Thus, this study illustrates the urgent need for coordinated local, regional, and international antimicrobial resistance surveillance efforts.

## Introduction

*Pseudomonas aeruginosa* is a Gram-negative bacterium with a diverse genetic composition allowing it to colonize various environments such as water, soil, and humans (Stover et al., 2000). It is a leading cause of nosocomial infections and can lead to many debilitating infections, including wound infections, pneumonia, and keratitis (Stapleton and Carnt, 2012). The success of *P. aeruginosa* as a human pathogen is due to (1) its arsenal of virulence factors that allow it to establish infections and survive under different conditions successfully, and (2) its ability to develop resistance to a wide variety of antibiotics through a myriad of methods, including efflux pumps, beta-lactamases, and porin channel mutations (Girlich et al., 2004). The numerous resistance mechanisms, particularly against last-resort drugs, make treating associated infections challenging, resulting in treatment failure, prolonged hospitalization, and high morbidity and mortality (Thuo et al., 2019). This penchant for developing multidrug resistance (MDR) and ability to cause life-threatening infections with high mortality rates (Farshadzadeh et al., 2014) earned *P. aeruginosa* the Priority 1: Critical designation among the World Health Organization’s (WHO) list of priority pathogens for antimicrobial research and development (World Health Organization, 2017).

*Pseudomonas aeruginosa* utilizes a variety of antibiotic resistance mechanisms, including multiple chromosomal determinants and complex regulatory pathways involved in adaptive and intrinsic resistance (Girlich et al., 2004). Compared to other Gram-negative bacteria, the innate MDR observed in *P. aeruginosa* is primarily due to inducible (MexXY) efflux pump production, expression of inducible AmpC cephalosporinase, lower outer membrane permeability, and production of the constitutive (MexAB-OprM) efflux pump. The reduction of natural susceptibility of *P. aeruginosa* to imipenem, aminopenicillins, and cephalosporins is mainly due to the expression of inducible AmpC by beta-lactamase production (Girlich et al., 2004). In addition, the acquisition of chromosomal mutations in the complex regulatory cell wall recycling pathways’ genes is a common mutation-driven mechanism for resistance. Some examples include the overproduction of chromosomal AmpC cephalosporinase and mutational inactivation of *ampD* (which encodes an N-acetylmuramyl-l-alanine amidase) and *dacB* (which encodes PBP4) genes (Moya et al., 2009). Fluoroquinolone resistance of *P. aeruginosa* is due to mutations in type IV topoisomerase (*ParC* and *ParE*) and DNA gyrase (*GyrA* and *GyrB*) genes in the quinolone resistance-determining regions (QRDR). Carbapenems are some of the last-resort drugs for treating Gram-negative bacterial infections. Carbapenemase enzymes (SME, IMI, NMC, GES, and KPC families) are common among *P. aeruginosa* (Thuo et al., 2019). The leading acquired resistance mechanism to carbapenems is the class B Metallo-β-lactamases (MBLs) such as GIM, AIM, SPM, IMP, and VIM. Despite IMP and VIM variants being reported worldwide, AIM, GIM, and SPM have been reported only in a few geographical areas like Germany and São Paulo (Zhao and Hu, 2010). The innate resistance of *P. aeruginosa* to some antibiotics, accumulation of mutations, and horizontally acquired resistance mechanisms have led to the global distribution of high-risk MDR clones such as ST111, ST175, and ST235 (Barrio-Tofiño et al., 2017) due to global human travel.

*Pseudomonas* bacteria establish infections using several essential virulence factors. For example, *P. aeruginosa* creates biofilms on the affected tissues’ surfaces, such as alginate (*alg*) and elastases (*lasA* and *lasB*), which disrupt tight junctions between host epithelial cells. *Pseudomonas* also utilize quorum-sensing (Q.S.) system proteins (*lasR/lasI* and *rhlR/rhlI*) which facilitate communication between cells and can use type III secretory proteins (*exoT*, *exoS*, *exoY*, and *exoU*) as well, which control the expression of exotoxins (Alonso et al., 2020). Other virulence factors inhibit the effects of antibiotics on *P. aeruginosa*. For example, the pyocyanin pigment enhances the alteration of mitochondrial electron transport of the host through oxidative stress, while pyoverdine upregulates the transcription of elastases (Gellatly and Hancock, 2013; Alonso et al., 2020).

There is a paucity of studies addressing the prevalence and epidemiology of *P. aeruginosa* in Kenya, which warrants redress, though existing research indicates that *P. aeruginosa* is a leading cause of nosocomial infections in the country (Pitout et al., 2008; Mutonga et al., 2019). Further, antimicrobial resistance in *P. aeruginosa* is a present threat in Kenya. A 2018 study at Kenyatta National Hospital reported a 60% prevalence of MDR *P. aeruginosa* among critical care patients and a 35% prevalence among outpatients (Mukaya et al., 2018), while a 2019 study by Wangai et al. (2019) reported a 30% prevalence of carbapenem resistance in *P. aeruginosa* in a large public tertiary hospital in Kenya. Additionally, Musila et al. (2021) found that of carbapenem-resistant *P. aeruginosa* isolates obtained from hospitals across Kenya, 13/14 were resistant to all antibiotics. However, the *P. aeruginosa* strains from Kenya have not been classified *via* multilocus sequence typing (MLST) or characterized in detail using whole-genome sequencing (WGS). This genetic and epidemiologic mapping is essential to establish the bacterium’s local and global transmission patterns and distribution. The WHO declared AMR one of the top 10 global public health threats facing humanity. It emphasized the importance of developing and maintaining robust surveillance and reporting systems for AMR epidemiology, genetics, and mechanics to combat AMR across the globe (World Health Organization, 2020). WGS is beneficial for the surveillance of drug resistance and virulence mechanisms to target treatment modalities tailored to different bacterial pathogens. Therefore, this study seeks to understand the phylogeny and genomic traits associated with the *P. aeruginosa* isolates from hospitals across Kenya to fill essential gaps in understanding the diversity of *P. aeruginosa* strains in the country.

## Materials and Methods

### Isolate Culture and Antimicrobial Susceptibility Testing

This study analyzed 56 clinical *P. aeruginosa* isolates obtained from study subjects with bacterial urinary tract (UTI; *n* = 9) and skin and soft tissue (SSTI; *n* = 47) infections recruited from an antimicrobial resistance surveillance study conducted between 2015 and 2020. *Pseudomonas aeruginosa* isolates were obtained from six hospitals within five counties in Kenya: Nairobi (27 isolates), Kisumu (15 isolates), Kilifi (seven isolates), Kisii (six isolates), and Kericho (one isolate). The Kisii and Nairobi county hospitals are referral and full-service teaching hospitals with large inpatient capacities. In contrast, the other four hospitals are county or sub-county level hospitals that offer inpatient, surgical, laboratory, maternity, and primary outpatient care services. Identification and automated phenotypic antimicrobial susceptibility testing (AST) of isolates were carried out on the VITEK 2® automated platform (bioMérieux, Marcy l’Etoile, France) using GN-ID and XN05 AST cards. Minimum inhibitory concentration (MIC) data of antibiotics (cefepime, ticarcillin-clavulanic acid, piperacillin, meropenem, levofloxacin, tetracycline, and tigecycline) for each isolate were interpreted for susceptibility and resistance according to the VITEK2® Advanced Expert System and the Clinical and Laboratory Standards Institute guidelines of 2017. MDR was defined as resistance to at least one agent in at least three antibiotic classes (Magiorakos et al., 2012).

### DNA Extraction and Whole-Genome Sequencing

Whole-genome sequencing was performed for 46 suspected MDR isolates on an Illumina MiSeq platform, as previously described by Musila et al. (2021). Briefly, total DNA was extracted using the DNeasy UltraClean Microbial Kit (Qiagen, Germantown, MD, United States) and paired-end libraries prepared using the KAPA HyperPlus Library preparation kit (Roche Diagnostics, Indianapolis, Indiana, United States) before quantification using the KAPA Library Quantification Kit–Illumina/Bio-Rad iCyclerTM (Roche Diagnostics, Indianapolis, Indiana, United States). Next, the libraries were sequenced with a MiSeq Reagent Kit v3 (600 cycles) on an Illumina MiSeq desktop sequencer (Illumina Inc., San Diego, CA, United States), after which species identification and contamination detection were performed from sequencing reads using the Kraken2 database (Wood and Salzberg, 2014).

Whole-genome sequencing was performed on an additional 10 suspected non-MDR isolates using the Oxford Nanopore MinION (Oxford Nanopore Technologies, Oxford, United Kingdom). Total DNA was extracted using the DNeasy UltraClean Microbial Kit (QIAGEN Inc., the Netherlands). Libraries were prepared with the NEBNext® Companion Module for Oxford Nanopore Technologies® Ligation Sequencing kit (New England Biolabs, Massachusetts, Unites States), Ligation Sequencing Kit SQK-LSK109 (Oxford Nanopore Technologies, Oxford, United Kingdom), and EXP-NBD 104 (1–12) Native Barcoding kit (Oxford Nanopore Technologies, Oxford, United Kingdom) according to the manufacturer’s instructions. All mixing steps for the DNA samples were performed by gently flicking the microfuge tube rather than pipetting. The pooled DNA library was quantified on the Qubit dsDNA fluorometer (ThermoFisher Scientific, Massachusetts United States) before loading onto a FLO-MIN106 R9.4.1 flowcell for sequencing based on the standard 1D sequencing protocol launched in the MinKNOW v20.10.3 software (Oxford Nanopore Technologies, Oxford, United Kingdom). Base-calling the raw MinION data and de-multiplexing of FastQ reads were completed using the Guppy software version 4.4.2 (Oxford Nanopore Technologies, Oxford, United Kingdom). The reads were filtered for quality using the default Q score of 8.

### Genomic Characterization, Strain Typing, and Phylogenetic Analysis

The long reads from Oxford Nanopore sequencing and the paired-end short reads from Illumina sequencing were quality assessed using the *NanoPack* (De Coster et al., 2018) and *FastQC v0.11.9* (Andrews, 2010), respectively. *Trimmomatic v0.39* (Bolger et al., 2014) was used to trim the adapters and low-quality sequences from the short reads before *de novo* assembly. The long reads were assembled using *Flye v2.8* (Kolmogorov et al., 2019), and the paired-end short reads were assembled using *Shovill v1.0.9* (Seemann, 2019). All assemblies were quality assessed using the *QUAST v5.0.2* (Gurevich et al., 2013). MLST assignment was performed on the assemblies using the command line *mlst v2.19* (Seemann, 2013) pipeline against the *P. aeruginosa* scheme hosted by the *PubMLST* database (Jolley et al., 2004) to determine the sequence types (ST).

#### Phylogenetic Analysis

The 56 assemblies were annotated using *Prokka v1.14.6* (Seemann, 2014), and the *gff3* output files were used to generate a core genome alignment using *Roary v3.13.0* (Page et al., 2015). The core genome phylogeny was inferred from the core genome alignment, and a maximum likelihood tree from the informative SNPs was constructed using *FastTree v2.1.10* (Price et al., 2010) based on the general time-reversible (GTR) model with 1,000 bootstrap replicates. The SNP distances (*p distances*) between clades were computed from the core genome alignment using MEGAX (Kumar et al., 2018) and the “compute between group mean distances” option. Seventy-five complete genomes of *P. aeruginosa* including reference strains PAO1, *P. aeruginosa* VRFPA04, *P. aeruginosa* UCBPP-PA14, *P. aeruginosa* PA7, *P. aeruginosa* LESB58, *P. aeruginosa* NCGM2-S1, and *P. aeruginosa* DK2 were downloaded from the GenBank database of NCBI and re-annotated using *Prokka v1.14.6* (Seemann, 2014) to avoid annotation bias. Combined with the 56 isolates from this study, the core genome phylogeny was inferred from the core genome alignment as described above. All trees were annotated with ST and geographical sources of isolates using the *ggtree v2.0* (Yu et al., 2017) and *ggplot v2.0* (Wickham, 2016) packages in R.

#### Resistance and Virulence Genes

Acquired resistance genes were identified from *de novo* assemblies using the *ABRicate v1.0.1* (Seemann, 2017) based on the *Comprehensive Antibiotic Resistance Database* (CARD; Alcock et al., 2020) and detection of known virulence genes was based on the *Virulence Factor Database* (VFDB; Chen et al., 2016). The genes prediction thresholds are 80% minimum DNA identity and 80% minimum DNA coverage. Resistance associated with chromosomal mutations in the QRDR were detected using *PointFinder v2.0* (Zankari et al., 2017). The detected genes were presented graphically as presence versus absence heatmaps using *Interactive Tree of Life (iTOL) v5.0* (Letunic and Bork, 2021).

### Study approvals and consent to participate

Ethical approval for the study was obtained from the Kenya Medical Research Institute (KEMRI) Scientific and Ethics Review Unit (#2767), the Walter Reed Army Institute of Research (WRAIR) Institutional Review Board (#2089), and the U.S. Army Medical Research and Materiel Command, Office of Research Protection, Human Research Protections Office (USAMRMC ORP HRPO) (Log#A-18129). The investigators adhered to the policies for the protection of human subjects as prescribed in AR 70–25. In addition, written informed consent was obtained from each participant before study enrollment.

## Results

### Genomic Features of *Pseudomonas aeruginosa* Isolates

The short paired-end reads from 46 isolates sequenced on the Illumina platform had on average total reads of 2.5 × 10^6^ with a mean quality Phred score of 35. In comparison, the long reads from 10 isolates sequenced on the Oxford Nanopore MinION platform had on average 2.0 × 10^3^ reads with a mean quality Phred score of 8. After assembly, the Illumina reads yielded assemblies with a mean coverage of 159x, an average number of 104 contigs, and a mean N50 of 572,756. In contrast, most assemblies from the Oxford Nanopore reads yielded a single circular chromosome with a mean coverage of 37x. All isolates had an average genome size of 6.6 Mbp with a mean G + C content of 66.4. A summary of the sources, sequencing platform and assembly characteristics for the 56 isolates studied is shown in Supplementary Table S1. All assemblies were uploaded to NCBI GenBank under the project number PRJNA580263.

### Multilocus Sequence Typing, Diversity, and Distribution

**Figure 1:**
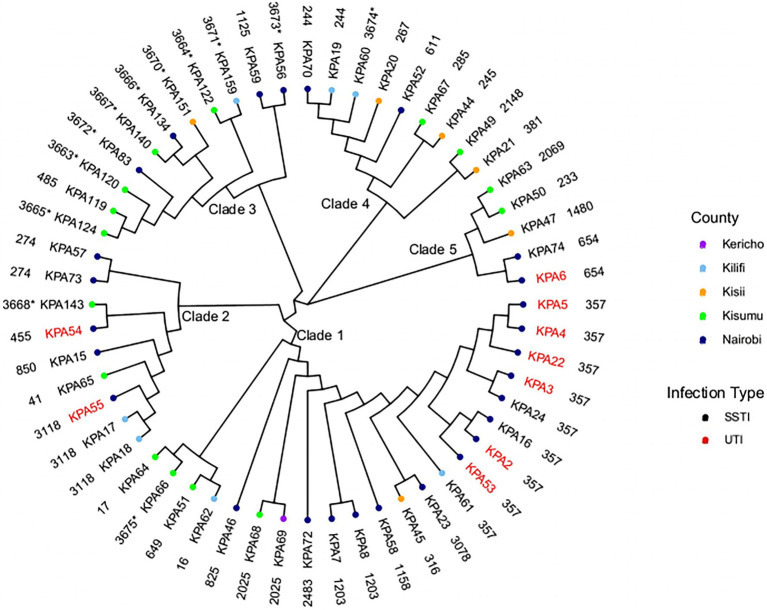
Phylogenetic analysis of Kenyan *Pseudomonas aeruginosa* isolates. A maximum-likelihood phylogenetic tree of 56 study isolates and their source regions. SSTI, skin and soft tissue infections; and UTI, urinary tract infections. The outer layer shows sequence types (ST) with the novel STs asterisked (*).

The fifty-six isolates belonged to 41 different STs (Figure 1). The most common ST was ST357 (nine isolates) followed by ST3118 (three isolates), ST1203, ST657, ST274, ST244, and ST2025 (each represented by two isolates), with the remaining 19 STs were represented by a single isolate. A maximum-likelihood tree was generated using the core genome alignment of the 56 isolates to obtain additional information regarding the phylogenetic relationships of the isolates (Figure 1). The isolates clustered according to STs resulting in five clades (Figure 1) distributed across all counties. The SNP distances among different clades are presented in Supplementary Table S2. There was a difference in the distribution of STs across counties with Kisumu (15 STs/15 isolates), Kericho (one ST/one isolate), and Kisii (six STs/six isolates), followed by Kilifi (six STs/7sevenisolates) and Nairobi (16 STs/27 isolates).

Several STs spanned different geographical regions. ST3118, ST357, and ST244 were found in Nairobi and Kilifi counties only. ST357 was present in eight isolates from Nairobi (KPA16, KPA2, KPA22, KPA24, KPA3 KPA4, KPA5, and KPA53) and one isolate from Kilifi county (KPA61), ST3118 presented in two isolates from Kilifi (KPA17 and KPA18) and one isolate from Nairobi (KPA55), and ST244 had one isolate from Kilifi (KPA19) and one from Nairobi (KPA70). ST2025 isolates were found in Kisumu (KPA68) and Kericho (KPA69). A single isolate represented each of the remaining seven STs except in Nairobi County, where two isolates each of the following STs were detected: ST1203 (KPA7 and KPA8), ST654 (KPA74, KPA6), and ST274 (KPA57 and KPA73). As the study isolates were predominantly from SSTIs (84%), the identified STs were associated with this infection type. However, the nine isolates recovered from UTI belonged to only four STs (ST654, ST357, ST55, and ST54), mostly ST357 (*n* = 6). Many isolates recovered from UTIs in the study were from inpatients (*n* = 9) with catheters at the Nairobi hospital suspected to have an outbreak of ST357 isolates. The other isolate collected from a UTI was associated with the MDR lineage ST654.

Sequences of 12 isolates with novel previously unassigned STs (KPA56, KPA60, KPA66, KPA83, KPA120, KPA122, KPA124, KPA134, KPA140, KPA143, KPA151, and KPA159) were submitted to the PubMLST database and assigned as ST3673, ST3674, ST3675, ST3672, ST3663, ST3664, ST3665, ST3666, ST3667, ST3668, ST3670, and ST3671, respectively, based on their allelic profiles (Table 1). The complete list of STs of the study isolates is in Supplementary Table S3.

**Table 1:** Allelic profiles of novel *Pseudomonas aeruginosa* multilocus sequence types (MLST).

Isolates	Sequence types	Allelic profiles of the housekeeping genes						
		*acsA*	*aroE*	*guaA*	*mutL*	*nuoD*	*ppsA*	*trpE*
KPA120	3663	11	5	6	229	2	164	211
KPA122	3664	98	3	17	5	2	10	268
KPA124	3665	16	259	19	3	4	121	201
KPA134	3666	6	5	58	11	2	164	201
KPA140	3667	219	3	6	13	2	153	201
KPA143	3668	243	5	12	11	2	158	201
KPA151	3670	1	5	149	3	2	25	201
KPA159	3671	6	299	168	3	2	164	201
KPA56	3673	28	5	7	3	2	6	7
KPA60	3674	17	5	12	3	2	4	172
KPA66	3675	11	6	6	3	2	76	7
KPA83	3672	17	5	11	5	2	38	201

**Figure 2:**
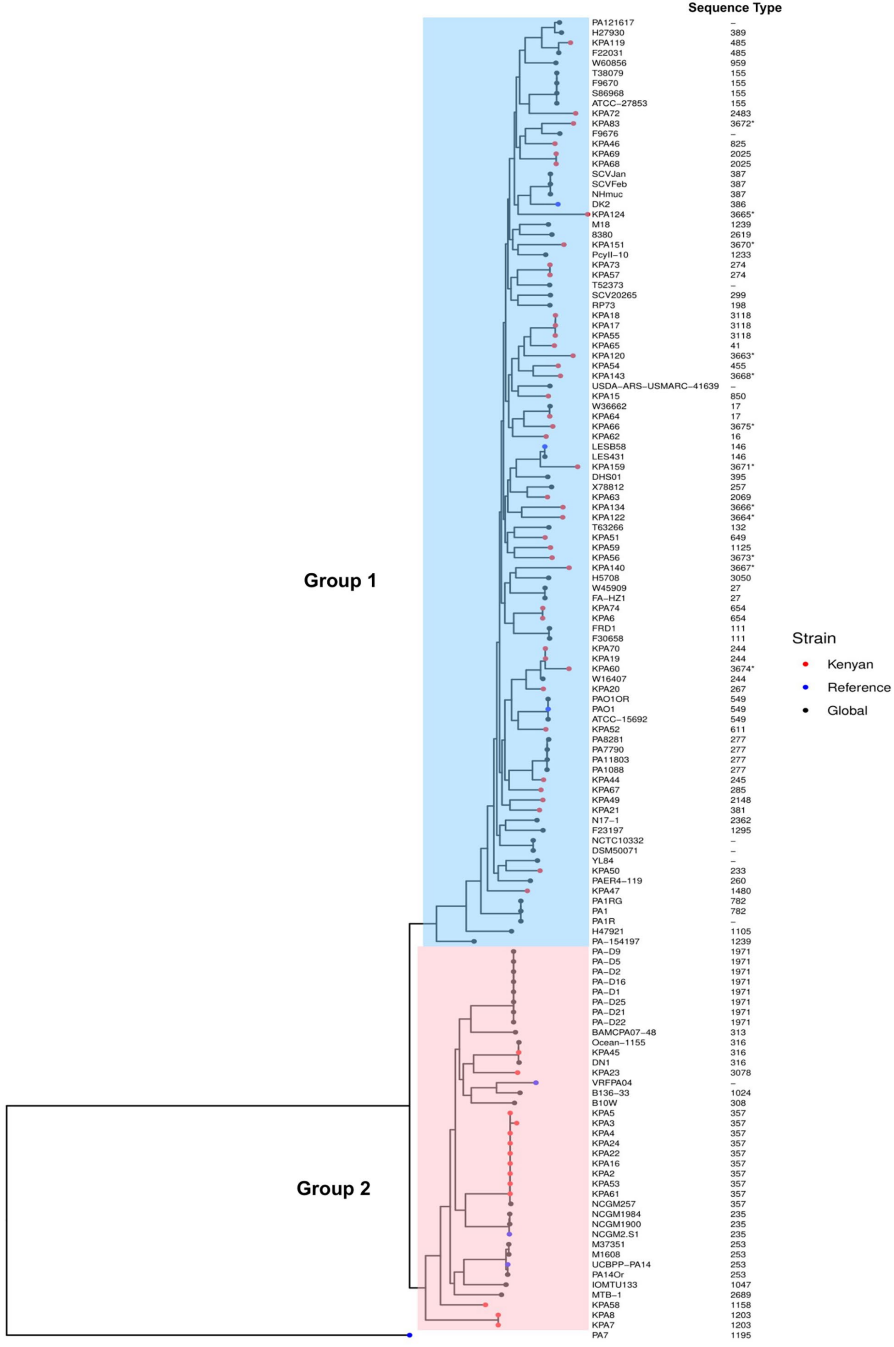
Phylogenetic analysis of Kenyan and global strains of *Pseudomonas aeruginosa*. A maximum-likelihood phylogenetic tree of all study isolates and global strains of *P. aeruginosa* with their sequence types, demonstrating two distinct groups. Group 1 isolates are shaded blue, and Group 2 are shaded pink. Strains used in this study are indicated as red dots, global strains are black dots, and global reference strains are blue dots (*P. aeruginosa* PAO1, *P. aeruginosa* VRFPA04, *P. aeruginosa* UCBPP-PA14, *P. aeruginosa* PA7, *P. aeruginosa* LESB58, *P. aeruginosa* NCGM2-S1, and *P. aeruginosa* DK2). The accession numbers of all genomes from GenBank used in this study are in Supplementary Table S2. The sequence types are indicated adjacent to the strains on the phylogenetic tree. Asterisks (*) depict novel sequence types.

A maximum-likelihood tree was created based on the core genome alignment of the 56 isolates and 75 global *P. aeruginosa* genomes obtained from the NCBI GenBank database. All strains clustered into two groups except the *P. aeruginosa* strain PA7, a taxonomic outlier included as an outgroup. Group 1 contained the majority 75% (42/56) of the study isolates and included several widely studied strains PAO1 (549), LESB58 (146), and DK2 (386). In contrast, Group 2 was smaller and contained local strains belonging to ST316, 357, and 1203 and the known virulent and MDR strain UCBPP-PA14 (ST253)—(Figure 2), mirroring the genetic diversity seen among *P. aeruginosa* globally. The complete list of the strains from the NCBI GenBank database and their allelic profiles are in Supplementary Tables S4, S5, respectively.

### Antibiotic Drug Resistance Profile and Resistance Genes

**Figure 3:**
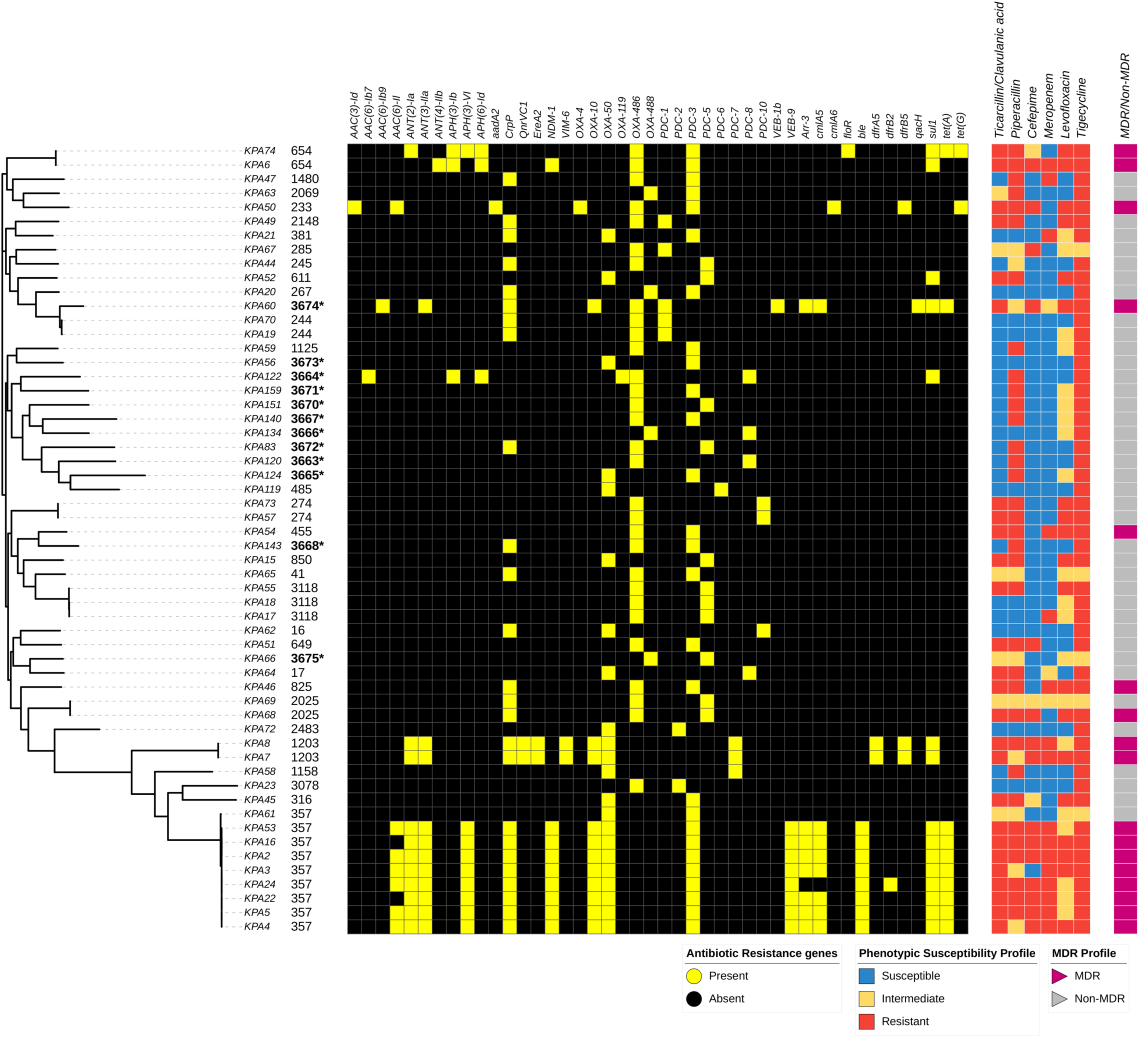
Distribution of sequence types, variably present antibiotic resistance genes, and susceptibility profiles among *Pseudomonas aeruginosa* isolates mapped on the maximum-likelihood phylogenetic tree of all study isolates, and the presence and absence of resistance genes as detected by the Comprehensive Antibiotic Resistance Database (CARD) is depicted. Associated resistance genes include: Beta lactamases: *PDC*, *NDM-1*, *OXA*, *VEB*, & *VIM-6*, Phenicols: *floR* & *cmlA*, Glycylcyclines: *tet*, Sulfonamides: – *sul*, Aminoglycosides: *aac*, *aph*, & *ant*, Fluoroquinolones: – *QnrVC1* & *crpP*, Macrolides: *EreA*, Trimethoprim: – *dfrB*, Glycopeptide: *ble*. Isolates with an asterisk (*) depict novel strains.

Antimicrobial susceptibility testing determined that 79% (44/56) of the isolates were non-susceptible to piperacillin, 57% (32/56) were non-susceptible to ticarcillin/clavulanic acid, 70% (39/56) were non-susceptible to levofloxacin, 34% (19/56) were non-susceptible to meropenem, and 32% (18/56) were non-susceptible to cefepime. Three isolates (KPA6, KPA2, and KPA16) were non-susceptible to all six antibiotics tested, while four isolates (KPA50, KPA54, KPA46, and KPA68) were susceptible to only one antibiotic. Six isolates (KPA70, KPA20, KPA56, KPA119, KPA62, and KPA72) were non-susceptible only to tigecycline, and no isolates were susceptible to tigecycline, as shown in Figure 3. Antibiotic susceptibility testing demonstrated that 31% (17/56) of the isolates were MDR, of which 47% (8/17) belonged to ST357. Of the isolates with novel STs, one isolate (KPA60) corresponding to novel ST3674 was MDR, whereas the other novel STs were non-MDR. The complete details of the antimicrobial susceptibility tests results are in Supplementary Table S6.

Genomic analysis identified 90 resistance genes, of which 46 (51%) were present in all isolates. These resistance genes were *Mex*, *Mux*, *Opm*, and *Opr* from the Mex-Opm-Opr operon, *fosA*, *CatB7*, *A.P.H. (3′)-Ilb*, *arnA*, and *tri* group genes. The other 44 (49%) resistance genes were variably present among the isolates. These were associated with different antibiotic classes such as *bla*PDC, *bla*NDM, *bla*OXA, *bla*VEB, *bla*NDM, and *bla*VIM for beta-lactams; *floR* and *cmlA* for phenicols; *tet(A)* and *tet(G)* for Glycylcyclines; *sul* for sulfonamides; *aac*, *aph*, and *ant* groups for aminoglycosides; *QnrVC1* and *crpP* for fluoroquinolones; *EreA* for macrolides; *dfrB* groups for trimethoprim; and *ble* for glycopeptide bleomycin. The ST357 lineage carried the greatest number of resistance genes. Notably, the carbapenemase genes *bla*NDM-1 were associated with ST654 and ST357 isolates, whereas *bla*VIM-6 was associated with ST1203. The resistance genes found variably among isolates and different STs are shown in Figure 3. The complete list of detected resistance genes is in Supplementary Table S7.

Analysis of the QRDR found that among the 56 isolates, 14 (25%) had mutations in *gyrA* while 22 (39%) had mutations in *parC*. All isolates with *gyrA* mutations also had *parC* mutations, whereas 10 (18%) isolates had mutations in *parC* only. About 15 mutations in *gyrA* resulted in an amino acid change at codon 83 (Threonine to Isoleucine), while 13 mutations in the *parC* gene resulted in an amino acid change at codon 87 (Serine to Leucine). In addition, several novel missense mutations were detected in *gyrA*: Asp 652 Tyr (one isolate) and in *parC* gene: Thr 556 Ser (two isolates), Val 419 Leu (five isolates), Pro 752 Thr (11 isolates), Val 646 Leu (seven isolates), and His 262 Gln (two isolates). The detailed list of the mutations in QRDR is in Supplementary Table S6.

### Virulence Genes

**Figure 4:**
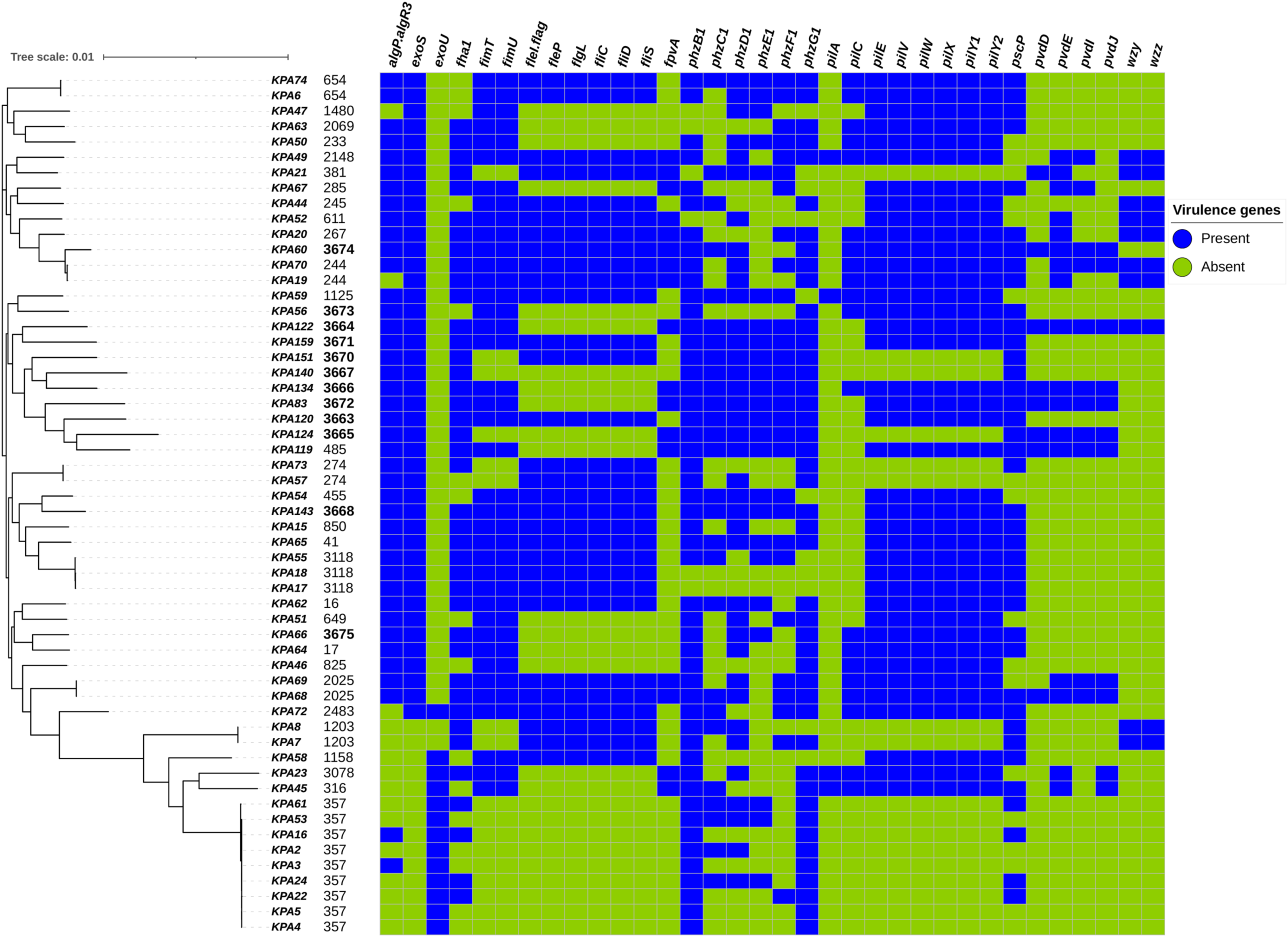
Distribution of variably present virulence genes among *Pseudomonas aeruginosa* isolates mapped on the maximum-likelihood phylogenetic tree of all study isolates, and the presence and absence of virulence genes as detected by the Virulence Factor Database (VFDB) are illustrated.

A total of 242 virulence genes were identified, of which 208/242 (86%) were present in all isolates, and 34/241 (14%) were variably present among isolates. All strains contained at least three of the 34 variable virulence genes, which fell into the functional categories of type II and IV secretory systems, flagellar and pilus biogenesis, alginate, phenazine, pyoverdine, and O-specific antigen biosynthesis. The presence of variable virulence genes among isolates and different sequence types is illustrated in Figure 4, and the complete list of detected virulence genes can be found in Supplementary Table S8.

In general, the relationship between virulence factors and phylogeny was more distinctive than with other factors such as drug resistance, clinical presentation, or geography. Isolates corresponding to ST357 (KPA2, KPA4, and KPA5) exhibited the fewest number of virulence genes at 87% (211/242), while the ST3674 isolate KPA60 contained the highest number of virulence genes at 98% (236/242). Among the variably present virulence genes, *phzB1* was most abundant, being present in 89% (50/56) of the isolates. STs with the most phenazine (*phz*) genes were the endemic novel strains (ST3664, 3671, 3670, 3667, 3666, 3672, 3663, 3665, 3668) and several previously identified STs (ST485, 41, and 654). In contrast, the pyoverdine synthesis genes (*pvd*) were not common and were present in only 15 STs. *wzy* and *wzz* were the least abundant virulence genes, present in only 18% (10/56) of the isolates. Around 21% (12/56) of the isolates contained an *exoU* gene and lacked an *exoS* gene, while 73% (41/56) of the isolates contained an *exoS* gene and lacked an *exoU* gene. Isolate KPA72 (ST 2483) contained both *exoU* and *exoS* genes, while isolates KPA7 and KPA8 (corresponding to ST 1203) lacked both *exoU* and *exoS* genes.

Other virulence genes identified included flagellar genes (*fleI/flag*, *fleP*, *flgL*, *fliC*, *fliD*, and *fliS*) and pilin genes (*fimT*, *fimU*, *pilE*, *pilV*, *pilW*, *pilX*, *pilY1*, and *pilY2*). Notably, ST357, ST3665, and ST3667 were the only STs lacking flagellar and pilli genes. Pilus protein genes were present in all but a few STs (ST381, ST3670, ST3667, ST3665, ST274, ST1203, and ST357), whereas flagellar proteins were present in a greater number of STs. Genes regulating alginate exopolysaccharide production (algP.algR3) and biofilm formation were widespread and absent in only ST1480, ST244, and a phylogenetically similar cluster of STs (2483, 1203, 1158, 3078, 316, and 357).

## Discussion

Our study identified 41 STs among the *P. aeruginosa* isolates, which is consistent with other studies that have reported the non-clonal population structure of *P. aeruginosa* (Barrio-Tofiño et al., 2020). The isolates from all regions exhibited this high genetic diversity except Nairobi County, which demonstrated a clonal distribution suggestive of local outbreaks in one hospital over the study period. Isolate KPA60 from Kilifi County (associated with the novel ST3674) was MDR and presented with an abundance of virulence genes, unlike isolates from other novel STs identified. Thus, this novel ST3674 is a clone that should be closely monitored.

The most prevalent ST among the isolates, MDR lineage ST357, is among the most globally dominant high-risk clones reported in many countries, including India (Kos et al., 2015). It is primarily associated with O-antigen serotype O11, *exoU*+/*exoS*− T3SS genotype, and MBLs such as VIM-2, IMP-1,6, and OXA-1,2,10 (Mano et al., 2015; Oliver et al., 2015; Miyoshi-Akiyama et al., 2017). In addition, another globally recognized highly resistant and virulent clone, ST654, was detected among the study isolates. ST654 is associated with the O-antigen serotype O11 and MBLs, including VIM-2 and OXA-1. However, in contrast to ST357, ST654 is linked to the T3SS genotype (*exoS*+ and *exoU*−) and other MBLs like NDM-1, and it has been identified in the United Kingdom, Korea, and Singapore (Wright et al., 2015; Zowawi et al., 2018; Barrio-Tofiño et al., 2020). The dominance of these high-risk clones, ST357 and ST654, in Nairobi County (at the capital of Kenya) and Kilifi County (at the coast) indicate that they are likely imported from other countries and could be associated with potential outbreaks in Kenya.

The phylogenetic analysis showed isolates clustering together into two major groups. The more extensive Group 1 includes the reference strain PAO1 (Stover et al., 2000), while the smaller Group 2 contains known MDR and virulent strains such as VRFPA04 (Murugan et al., 2016) and UCBPP-PA14 (Lee et al., 2006) in concurrence with other studies (Stewart et al., 2014; Subedi et al., 2018). The grouping into two major groups confirmed studies that indicate a close genetic relatedness among human *P. aeruginosa* strains in contrast to the greater diversity observed among environmental strains (Winstanley et al., 2009). All isolates belonging to ST357 clustered in Group 2 were closely related to the NCGM257 strain (ST357) isolated from a patient with a UTI in Tokyo, Japan. High-risk endemic clones ST1203, ST244, and ST233 (Barrio-Tofiño et al., 2020) have emerged independently (KPA7/8) or from Asia and the United States based on closest neighbors’ analysis. This implied global fluidity in the movement of strains from Asia and the United States would account for the carbapenemases VIM-6 and NDM-1 associated with ST1203 and ST357/ST654, respectively, in the Kenyan isolates. Most MDR strains were detected at the main entry points into the country, Nairobi, and the Indian Ocean coast, which suggests that importation is a likely mechanism of introducing MDR strains into Kenya’s *P. aeruginosa* population. In contrast, endemic strains were relatively non-MDR.

The most effective antibiotic regimen for *P. aeruginosa* is a combination of an anti-pseudomonal beta-lactam (e.g., piperacillin), a carbapenem, or a fluoroquinolone (Bassetti et al., 2018). From this study, the antimicrobial resistance patterns of the *P. aeruginosa* isolates to levofloxacin and meropenem were 31 and 30%, respectively. Although these levels are lower than the 61% of levofloxacin and 54% of meropenem resistance that were found in a study by Mukaya et al. (2018) at Kenyatta National Hospital (a large referral hospital in Kenya), the high levels of piperacillin resistance in both studies (63 and 56.7%, respectively) suggests a reduced efficacy in the combination treatment for *P. aeruginosa*. Furthermore, the growing resistance patterns highlight the grave public health threat MDR *P. aeruginosa* poses.

This study identified a prevalence of 31% MDR strains among study isolates of *P. aeruginosa*, a marked increase from the 13.7% prevalence of MDR *P. aeruginosa* reported in a 2008 study at Aga Khan University Hospital in Kenya (Pitout et al., 2008). The local increase in prevalence reflects the growing global rise in MDR *P. aeruginosa*, as reflected in the 26% prevalence of MDR *P. aeruginosa* infections reported in a 2017 nationwide survey in Spain (Barrio-Tofiño et al., 2019). MDR strains are associated with high morbidity and mortality rates, particularly in immunocompromised patients (Ozgumus et al., 2007), which has negative clinical and economic implications due to increased all-cause mortality and extended hospital stays (Nathwani et al., 2014).

The high MDR prevalence in this study is possibly attributed to chromosomal mutations and integrons carrying genes conferring resistance to aminoglycosides, β-lactams, quinolones, among others (Jeong et al., 2009). The MBL carbapenemase genes *VIM-6* and *NDM-1* (identified in ST654, ST1203, and ST357 study isolates) were recently reported in our published work among *P. aeruginosa* isolates in Kenya (Musila et al., 2021). *NDM-1* carbapenemases are unusual for ST357 isolates and are typically associated with IMPs and VIM-2 (Oliver et al., 2015). The study also identified a rare fluoroquinolone resistance gene (*QnrVC1*) typically seen on a plasmid (Pv et al., 2019) but here appeared on the chromosome. The most frequent mutations in the QRDR were in *gyrA* and *parC* genes with hotspots at Thr83 (*n* = 14, 25%) and Ser87 (*n* = 13, 23%), respectively. The mutations have a high correlation with increased MICs to fluoroquinolone antibiotics like ciprofloxacin, as previously reported (Salma et al., 2013; Van Nguyen et al., 2018; Park et al., 2020). The novel mutations found in the QRDR were mainly in the *parC* gene and likely contributed to the high levofloxacin resistance among the *P. aeruginosa* isolates. Because fluoroquinolones and carbapenems are important for treating nosocomial infections, these novel resistance mechanisms increase the scope and potential for widespread antibiotic resistance to critical drugs.

Of the many virulence genes detected, 86% were found among all *P. aeruginosa* isolates, including endemic and novel strains, and were associated with biofilm formation, adhesion, and motility. Although most of the endemic strains were non-MDR, the number of virulence genes identified contributes to the establishment of persistent and drug-tolerant infections. For example, the production of the iron siderophores pyoverdine (fluorescein) and phenazine (Wang et al., 2011) enhance the persistence of infection in low iron conditions and biofilm formation. The flagellar, pilli, and alginate regulating genes enable attachment of the bacterium to the cell, enhance adhesion to cells, increase swimming motility across solid surfaces, and promote biofilm formation. Biofilms provide a mechanical barrier to antibiotics, whereas phenazines can promote antibiotic tolerance to ciprofloxacin in biofilms (Schiessl et al., 2019). These functions protect the bacteria from adversity and allow them to persist in adverse and hostile environments, thereby increasing their pathogenic potential.

Many studies have highlighted the interplay between virulence and antibiotic resistance. AMR is hypothesized to impose a biological cost resulting in compromised virulence of the bacteria, though whether resistance has a negative or positive effect on virulence remains debatable (Skurnik et al., 2013; Sawa et al., 2014). This biological compromise is evident in the most studied virulence factor of *P. aeruginosa*, the type III secretion system. This system injects ExoU, ExoS, ExoT, or ExoY cytotoxins into human cells, which determines the extent of host tissue injury. The *exoU* and *exoS* genes are often mutually exclusive, and their presence vs. absence pattern is associated with specific STs and their virulence levels (Hauser, 2009; Oliver et al., 2015). A study in Spain reported that the *exoS*+/*exoU*− type III secretion system genotype was positively associated with high-risk MDR clones (Peña et al., 2015). The current study identified one exoU+/exoS+ isolate ST2483 (KPA72), which is rare, as a mutual exclusion exists (Hauser, 2009; Juan et al., 2017). Furthermore, this isolate was susceptible to all antibiotics tested and contained minimal resistance genes, contrary to the study by Horna et al. (2019) that associated this genotype (*exoU+/exoS+*) with enhanced antibiotic resistance. Thus, this isolate appears to exhibit a trade-off between high virulence and antibiotic resistance.

One of the strengths of this study is its multicenter approach to exploring the epidemiology and genomics of clinical *P. aeruginosa*, which offers an improved understanding of the distribution of sequence types, antibiotic resistance patterns, and virulence of the bacterium in Kenya. This approach has mapped and monitored high-risk clones such as ST357, ST654, ST244, and the novel ST3674 across different regions in Kenya. A second strength is the use of Oxford Nanopore and Illumina sequencing technology which provided greater genomic resolution and clearer insight into the intrinsic antibiotic resistance and virulence characteristics of *P. aeruginosa*, as no plasmids were detected in the isolates. However, there were several limitations to the study as well. First, the coverage and quality of Oxford Nanopore sequences were low compared to the sequences from Illumina technology. Second, the epidemiological results from some regions, such as Kericho County, were inconclusive due to the limited number of isolates studied. Finally, the connection between the study genomes and the international high-risk clones showed limited variability, suggesting increased geographical dissemination and clonal expansion of these lineages in Kenya. This inability to link local with global strains could be due to limited representation of the East African region *P. aeruginosa* genomes in the collection of global genomes, highlighting the need for more studies on *P. aeruginosa* genomics in Kenya and East Africa as a whole. The whole-genome sequence data available in the GenBank database of NCBI from this study has contributed to closing this knowledge gap.

This study uncovered high-risk clones in Kenya, particularly in Nairobi County. It demonstrated that these clones likely emerged from the importation of international epidemic clones, and the study also illustrated the emergence of local endemic and virulent MDR strains with unique characteristics. These findings highlight the importance of MDR surveillance initiatives in providing regionally relevant data to hospitals on efficacious treatments for *P. aeruginosa*, identifying high-risk clones to monitor and detect outbreaks, and recognizing virulence factors that could impact the clinical course of infections. More extensive genomic studies on isolates from a wider range of hospitals across Kenya linked to the Kenya National AMR Surveillance Program (CDC, 2018) would benefit the management and help curb the rise of *P. aeruginosa* infections. In addition, this collective data would contribute to the global understanding of AMR transmission in support of the WHO AMR surveillance program (World Health Organization, 2014).

## Data Availability Statement

The datasets presented in this study can be found in online repositories. The names of the repository/repositories and accession number(s) can be found in the article/Supplementary Material.

## Ethics Statement

The studies involving human participants were reviewed and approved by Kenya Medical Research Institute (KEMRI) Scientific and Ethics Review Unit (#2767) and the Walter Reed Army Institute of Research (WRAIR) Institutional Review Board (#2089). Written informed consent to participate in this study was provided by the participants’ legal guardian/next of kin.

## Author Contributions

SK, CKy, and LM conceived and designed the study. SK, CKy, and AM performed the study. SK analyzed the data. SK, CKy, HS, and LM wrote the paper. SK, CKy, AM, HS, EM, GM, CKi, and LM reviewed the manuscript. All authors contributed to the article and approved the submitted version.

## Funding

This work was funded by the Fogarty International Center of the National Institutes of Health under Award Number U2RTW010677 and the Armed Forces Health Surveillance Division, Global Emerging Infections Surveillance (GEIS) Branch (PROMIS ID 20160270153 FY17-20). The content is solely the authors’ responsibility and does not necessarily represent the official views of the funders.

## Author Disclaimer

The material for this publication has been reviewed and approved by the Walter Reed Army Institute of Research, and there is no objection to its publication. The opinions or assertions contained herein are the private views of the authors and are not to be construed as official or reflecting the views of the Department of the Army or the Department of Defense. The investigators have adhered to the policies for the protection of human subjects as prescribed in AR 70-25.

## Conflict of Interest

The authors declare that the research was conducted in the absence of any commercial or financial relationships that could be construed as a potential conflict of interest.

## Publisher’s Note

All claims expressed in this article are solely those of the authors and do not necessarily represent those of their affiliated organizations, or those of the publisher, the editors and the reviewers. Any product that may be evaluated in this article, or claim that may be made by its manufacturer, is not guaranteed or endorsed by the publisher.
