# Supplementary material for: Genetic Diversity, Distribution, and Genomic Characterization of Antibiotic Resistance and Virulence of Clinical Pseudomonas aeruginosa Strains in Kenya
Source: Front Microbiol. 2022 Mar 14;13:835403. doi: 10.3389/fmicb.2022.835403 (PMC8964364; doi:10.3389/fmicb.2022.835403)
Supplement: Supplementary file 1 [file Data_Sheet_1.PDF]

Supplementary Table S1. Genomic characteristics of the 56 *P. aeruginosa* isolates

Isolate ID	Number of Contigs	Coverage	N50	GC (%)	Genome Length (bp)
KPA119*	1	40	6,368,373	66	6,368,373
KPA120*	1	35	6,345,918	66	6,345,918
KPA122*	2	55	6,500,926	67	7,019,499
KPA124*	1	25	6,262,914	67	6,262,914
KPA134*	1	38	6,335,889	67	6,335,889
KPA140*	1	29	6,309,268	67	6,309,268
KPA143*	1	18	6,634,608	66	6,634,608
KPA151*	1	33	6,374,920	67	6,374,920
KPA159*	1	28	6,407,102	66	6,407,102
KPA83*	1	49	6403424	66	6,403,424
KPA15	59	120	228,383	66	6,341,318
KPA16	105	90	140,800	66	6,672,575
KPA17	92	88	203,970	66	6,461,661
KPA18	87	150	191,350	66	6,455,972
KPA19	94	99	158,457	66	6,600,183
KPA2	111	230	160,768	66	6,668,619
KPA20	81	98	178,025	66	6,581,582
KPA21	83	92	165,717	66	6,616,132
KPA22	105	89	183,093	66	6,667,831
KPA23	90	94	159,966	67	6,279,590
KPA24	66	140	313,140	66	6,679,445
KPA3	151	160	86,930	66	6,617,116
KPA4	147	155	92,797	66	6,612,656
KPA44	168	120	73,503	66	6,893,836
KPA45	102	99	117,865	67	6,329,353
KPA46	101	130	139,819	66	6,356,917
KPA47	75	125	180,119	66	6,413,116
KPA49	102	100	136,319	66	6,450,307
KPA5	145	210	99,140	66	6,652,204
KPA50	107	170	137,831	66	6,696,701
KPA51	99	180	137,831	67	6,219,333
KPA52	84	165	184,527	66	6,370,522

KPA53	126	110	116,838	66	6,669,864
KPA54	126	120	93,277	66	6,484,783
KPA55	44	95	395,470	66	6,460,267
KPA56	86	98	156,925	67	6,216,266
KPA57	115	148	105,863	67	6,326,725
KPA58	152	105	80,329	66	6,456,720
KPA59	130	200	103,643	66	6,405,240
KPA6	77	185	245,737	66	6,888,511
KPA60	52	162	388,626	66	6,828,051
KPA61	38	178	423,690	66	6,695,968
KPA62	22	149	614,783	67	6,307,974
KPA63	32	166	769,266	67	6,110,399
KPA64	22	137	917,616	67	6,259,183
KPA65	31	153	655,270	66	6487603
KPA66	32	109	518,586	66	6,297,353
KPA67	42	182	622,417	67	6,265,832
KPA68	50	194	409,317	66	6,562,746
KPA69	59	126	408,991	66	6,554,859
KPA7	58	135	364,782	66	6,906,695
KPA70	38	155	468,965	66	6,559,748
KPA72	26	194	1,074,168	66	6,619,959
KPA73	26	182	783,245	66	6,353,136
KPA74	185	100	71,343	66	6,770,679
KPA8	62	195	285,146	66	6,905,418
**N50** - The sequence length of the shortest contig at 50% of the total genome length. The isolates sequenced on the Oxford Nanopore MinION platform are marked with an asterisk (*).					

Supplementary Table S2: SNP matrix showing SNP distance between the different clades on the Phylogenetic tree of the 56 study isolates.

Clade	1	2	3	4	5
1	-	0.0025399	0.0028975	0.002551	0.0025144
2		-	0.0018275	0.001482	0.0015211
3			-	0.0018802	0.0019011
4				-	0.001518
5					-
The figures in the SNP matrix of the clades are *p-distance.* This distance is the proportion (p) of nucleotide sites at which the two sequences compared are different. This is obtained by dividing the number of SNPs (n_d) by the total number of nucleotides compared (n). Thus, $p = n_d/n$. The p-distance is approximately equal to the number of nucleotide substitutions per site (d) only when it is small, that is $p < 0.1$.					

Supplementary Table S3. County, infection type, sequence type and multi-locus sequence allelic profiles of the *P. aeruginosa* isolates

Isolate	County	Infection Type	ST	Allelic Profiles of the Housekeeping Genes						
				acsA	aroE	guaA	mutL	nuoD	ppsA	trpE
KPA119	Kisumu	SSTI	485	11	76	5	3	61	14	3
KPA120	Kisumu	SSTI	3663	11	5	6	229	2	164	211
KPA122	Kisumu	SSTI	3664	98	3	17	5	2	10	268
KPA124	Kisumu	SSTI	3665	16	259	19	3	4	121	201
KPA134	Nairobi	SSTI	3666	6	5	58	11	2	164	201
KPA140	Kisumu	SSTI	3667	219	3	6	13	2	153	201
KPA143	Kisumu	SSTI	3668	243	5	12	11	2	158	201
KPA151	Kisii	SSTI	3670	1	5	149	3	2	25	201
KPA159	Kilifi	SSTI	3671	6	299	168	3	2	164	201
KPA83	Nairobi	SSTI	3672	17	5	11	5	2	38	201
KPA15	Nairobi	SSTI	850	11	5	6	3	4	4	19
KPA16	Nairobi	SSTI	357	2	4	5	3	1	6	11
KPA17	Kilifi	SSTI	3118	6	5	11	11	4	4	193
KPA18	Kilifi	SSTI	3118	6	5	11	11	4	4	193
KPA19	Kilifi	SSTI	244	17	5	12	3	14	4	7
KPA2	Nairobi	UTI	357	2	4	5	3	1	6	11
KPA20	Kisii	SSTI	267	19	5	12	11	11	4	14
KPA21	Kisii	SSTI	381	11	20	1	65	4	4	10
KPA22	Nairobi	UTI	357	2	4	5	3	1	6	11
KPA23	Nairobi	SSTI	3078	18	4	134	33	1	6	4
KPA24	Nairobi	SSTI	357	2	4	5	3	1	6	11
KPA3	Nairobi	UTI	357	2	4	5	3	1	6	11
KPA4	Nairobi	UTI	357	2	4	5	3	1	6	11
KPA44	Kisii	SSTI	245	39	6	12	11	3	15	2
KPA45	Kisii	SSTI	316	13	8	9	3	1	6	9
KPA46	Nairobi	SSTI	825	1	5	36	5	2	42	7

Isolate	County	Infection Type	ST	Allelic Profiles of the Housekeeping Genes						
KPA47	Kisii	SSTI	1480	11	10	94	3	1	12	26
KPA49	Kisumu	SSTI	2148	17	20	6	34	4	6	19
KPA5	Nairobi	UTI	357	2	4	5	3	1	6	11
KPA50	Kisumu	SSTI	233	16	5	30	11	4	31	41
KPA51	Kisumu	SSTI	649	11	84	11	3	4	4	7
KPA52	Nairobi	SSTI	611	17	5	12	98	4	14	10
KPA53	Nairobi	UTI	357	2	4	5	3	1	6	11
KPA54	Nairobi	UTI	455	6	14	12	11	1	4	20
KPA55	Nairobi	UTI	3118	6	5	11	11	4	4	193
KPA56	Nairobi	SSTI	3673	28	5	7	3	4	6	7
KPA57	Nairobi	SSTI	274	23	5	11	7	1	12	7
KPA58	Nairobi	SSTI	1158	5	4	3	6	1	33	42
KPA59	Nairobi	SSTI	1125	11	5	11	13	3	4	1
KPA6	Nairobi	UTI	654	17	5	26	3	4	4	26
KPA60	Kilifi	SSTI	3674	17	5	12	3	14	4	172
KPA61	Kilifi	SSTI	357	2	4	5	3	1	6	11
KPA62	Kilifi	SSTI	16	28	5	12	11	27	1	44
KPA63	Kisumu	SSTI	2069	35	5	36	72	4	42	1
KPA64	Kisumu	SSTI	17	11	5	1	7	9	4	7
KPA65	Kisumu	SSTI	41	28	5	6	11	4	4	14
KPA66	Kisumu	SSTI	3675	11	6	6	3	4	76	7
KPA67	Kisumu	SSTI	285	16	22	6	74	2	41	2
KPA68	Kisumu	SSTI	2025	11	196	12	34	4	13	18
KPA69	Kericho	SSTI	2025	11	196	12	34	4	13	18
KPA7	Nairobi	SSTI	1203	33	1	25	6	6	7	5
KPA70	Nairobi	SSTI	244	17	5	12	3	14	4	7
KPA72	Nairobi	SSTI	2483	25	4	11	3	3	6	8
KPA73	Nairobi	SSTI	274	23	5	11	7	1	12	7
KPA74	Nairobi	SSTI	654	17	5	26	3	4	4	26
KPA8	Nairobi	SSTI	1203	33	1	25	6	6	7	5

Isolate	County	Infection Type	ST	Allelic Profiles of the Housekeeping Genes
**ST –** Sequence Type				

Supplementary Table S4: Strains of *Pseudomonas aeruginosa* used in the study downloaded from NCBI GenBank database based on the availability of complete genome

Strain	Number of Genes	GenBank Accession	Source Country	Isolation Source
PAO1 (Reference)	5,697	NC_002516.1	USA	Wound
UCBPP-PA14	5,893	NC_008463.1	Brazil	burn wound
PA7	6,084	NC_009656.1	USA	non-respiratory clinical
LESB58	6,030	NC_011770.1	United Kingdom	sputum
X78812	5,782	CP008872.2	USA	missing
F9670	5,987	CP008873.1	USA	missing
F22031	6,077	CP007399.1	USA	pubic bone
NCTC10332	5,704	LN831024.1	Czech Republic	not known
DSM50071	5,803	CP012001.1	Japan	missing
S86968	6,360	CP008865.2	USA	missing
T38079	6,137	CP008866.2	USA	missing
T52373	5,643	CP008867.1	USA	missing
T63266	5,880	CP008868.1	USA	missing
W16407	6,181	CP008869.2	USA	missing
W36662	6,237	CP008870.2	USA	missing
W45909	6,225	CP008871.2	USA	missing
W60856	6,333	CP008864.2	USA	missing
F23197	5,905	CP008856.2	USA	missing
F30658	6,622	CP008857.1	USA	missing
H5708	5,804	CP008859.2	USA	missing
H27930	5,964	CP008860.2	USA	missing
H47921	6,249	CP008861.1	USA	missing
M1608	5,887	CP008862.2	USA	missing
M37351	6,322	CP008863.1	USA	missing
USDA-ARS-USMARC-41639	5,814	CP013989.1	USA	nasopharynx
NCGM1984	6,399	AP014646.1	Japan	catheter
NCGM1900	6,354	AP014622.1	Japan	catheter
FRD1	6,179	CP010555.1	USA	sputum
Carb01 63	7,070	CP011317.1	Netherlands	environment
DHS01	6,608	CP013993.1	France	nose
NCGM257	6,628	AP014651.1	Japan	Midstream urine
8380	6,086	AP014839.2	Japan	environment
IOMTU 133	6,276	AP017302.1	Japan	Urinary catheter
F9676	5,827	CP012066.1	China	environment

Strain	Number of Genes	GenBank Accession	Source Country	Isolation Source
PA1088	6,196	CP015001.1	Brazil	urine
NHmuc	5,795	CP013479.1	Netherlands	missing
PA1RG	5,994	CP012679.1	China	hospital sewage
F63912	6,149	CP008858.2	USA	missing
N17-1	5,867	CP014948.1	China	soil
PAER4_119	6,083	CP013113.1	Poland	Lindberg collection
ATCC 27853	6,312	CP015117.1	Netherlands	missing
BAMCPA07-48	6,438	CP015377.1	USA	combat injury wound
PA121617	6,303	CP016215.1	China	sputum
PA_154197	6,041	CP017306.1	Hong Kong	missing
PA_D1	6,069	CP012585.1	China	sputum
PA_D2	6,066	CP012578.1	China	sputum
PA_D9	6,065	CP012580.1	China	sputum
PA_D16	6,086	CP012581.1	China	sputum
PA_D22	6,091	CP012583.1	China	sputum
SCVJan	5,794	CP013478.1	United Kingdom	murine model
PA_D25	6,080	CP012584.1	China	sputum
PA_D5	6,087	CP012579.1	China	sputum
PA_D21	6,063	CP012582.1	China	sputum
ATCC 15692	5,744	CP017149.1	China	Infected wound
FA-HZ1	6,147	CP017353.1	China	wastewater
M18	5,770	CP002496.1	China	missing
NCGM2.S1	6,274	AP012280.1	Japan	missing
PA14OR	5,971	LT608330.1	France	missing
PcyII-10	5,847	LT673656.1	France	missing
DK2	5,959	CP003149.1	Denmark	sputum
B136-33	5,904	CP004061.1	Taiwan	diarrhea
RP73	5,864	CP006245.1	Germany	missing
YL84	5,908	CP007147.1	Malaysia	compost
DN1	6,626	CP017099.1	China	soil
MTB-1	6,186	CP006853.1	India	t-HCH contaminated soil
SCV20265	6,380	CP006931.1	Germany	lung
VRFPA04	6,308	CP008739.2	India	corneal button
SCVFeb	5,794	CP013477.1	United Kingdom	murine model
LES431	6,091	CP006937.1	United Kingdom	missing
PA11803	6,565	CP015003.1	Brazil	bloodstream

Strain	Number of Genes	GenBank Accession	Source Country	Isolation Source
PAO1OR	5,757	LN871187.1	France	missing
PA8281	6,408	CP015002.1	Brazil	tracheal aspirate
PA1R	5,689	CP004055.1	China	missing
PA1	5,987	CP004054.2	China	missing
B10W	6,317	CP017969.1	USA	wastewater
Ocean_1155	6,481	CP022526.1	Pacific Ocean	Ocean

Supplementary Table S5. Sequence type and multi-locus sequence allelic profiles of the *P. aeruginosa* used in the study downloaded from NCBI GenBank database based on the availability of complete genome

**Isolate**	**ST**	**Allelic Profiles of the Housekeeping Genes**						
		acsA	***aroE***	***guaA***	***mutL***	***nuoD***	***ppsA***	***trpE***
8380	2619	17	5	1	3	4	4	3
ATCC15692	549	7	5	12	3	4	1	7
ATCC27853	155	28	5	36	3	3	13	7
B10W	308	13	4	5	5	12	7	15
B136-33	1024	2	4	24	3	1	6	25
BAMCPA07-48	313	47	8	7	6	8	11	40
Carb0163	111	17	5	5	4	4	4	3
DHS01	395	6	5	1	1	1	12	1
DK2	386	17	5	11	18	4	10	3
DN1	316	13	8	9	3	1	6	9
DSM500	-	175	5	1	11	3	6	7
F22031	485	11	76	5	3	61	14	3
F23197	1295	11	5	124	67	4	115	3
F30658	111	17	5	5	4	4	4	3
F63912	198	11	5	11	11	3	27	7
F9670	155	28	5	36	3	3	13	7
F9676	-	40	5	11	5	4	28	37
FA-HZ1	27	6	5	6	7	4	6	7
FRD1	111	17	5	5	4	4	4	3
H27930	389	17	22	5	3	1	14	3
H47921	1105	23	5	12	30	1	4	7
H5708	3050	4	5	6	3	4	4	19
IOMTU133	1047	18	8	5	5	1	6	4
LES431	146	6	5	11	3	4	23	1

Isolate	ST	Allelic Profiles of the Housekeeping Genes						
LESB58	146	6	5	11	3	4	23	1
M1608	253	4	4	16	12	1	6	3
M18	1239	16	5	1	3	4	15	7
M37351	253	4	4	16	12	1	6	3
MTB-1	2689	5	8	3	5	1	11	3
N17-1	2362	6	5	1	29	92	4	68
NCGM1900	235	38	11	3	13	1	2	4
NCGM2.S1	235	38	11	3	13	1	2	4
NCGM1984	235	38	11	3	13	1	2	4
NCGM257	357	2	4	5	3	1	6	11
NCTC10332	-	175	5	1	11	3	6	7
NHmuc	387	28	5	11	11	4	12	3
Ocean-1155	316	13	8	9	3	1	6	9
PA1088	277	39	5	9	11	27	5	2
PA11803	277	39	5	9	11	27	5	2
PA14OR	253	4	4	16	12	1	6	3
PA7790	277	39	5	9	11	27	5	2
PA_150577	1239	16	5	1	3	4	15	7
PA1	782	15	3	3	11	1	15	1
PA1RG	782	15	3	3	11	1	15	1
PA1R	782	15	3	3	11	1	15	1
PA7	1195	87	34	43	37	53	107	126
PA8281	277	39	5	9	11	27	5	2
PA_D16	1971	32	190	3	62	8	7	26
PA_D1	1971	32	190	3	62	8	7	26
PA_D21	1971	32	190	3	62	8	7	26
PA_D22	1971	32	190	3	62	8	7	26
PA_D25	1971	32	190	3	62	8	7	26

Isolate	ST	Allelic Profiles of the Housekeeping Genes						
PA_D2	1971	32	190	3	62	8	7	26
PA_D5	1971	32	190	3	62	8	7	26
PA_D9	1971	32	190	3	62	8	7	26
PAER4_119	260	14	5	10	7	4	13	7
PAO1OR	549	7	5	12	3	4	1	7
PAO1	549	7	5	12	3	4	1	7
PcyII-10	1233	17	5	11	3	4	6	7
RP73	198	11	5	11	11	3	27	7
S86968	155	28	5	36	3	3	13	7
SCV20265	299	17	5	36	3	3	7	3
SCVFeb	387	28	5	11	11	4	12	3
SCVJan	387	28	5	11	11	4	12	3
T38079	155	28	5	36	3	3	13	7
T52373	-	16	10	5	3	64	42	7
T63266	132	6	20	1	3	4	4	2
UCBPP-PA14	253	4	4	16	12	1	6	3
USDA-ARS-USMARC-41639	-	11	8	19	115	4	13	18
VRFPA04	-	32	13	24	13	1	6	25
W16407	244	17	5	12	3	14	4	7
W36662	17	11	5	1	7	9	4	7
W45909	27	6	5	6	7	4	6	7
W60856	959	6	5	11	7	3	70	19
X78812	257	35	24	36	11	4	15	14
YL84	-	40	5	30	5	3	33	14
ST: Sequence type								

Supplementary Table S6: Strains with Antibiotics susceptibility tests and QRDR Mutations

Strains	AST Summary	Antibacterial Susceptibility Tests						QRDR Mutations			
		Antibiotics						*gyrA* gene		*parC* gene	
		TCC	PIP	FEP	MEM	LVX	TIG	NC	AC	NC	AC
KPA55	Non-MDR	**R**	**R**	S	S	**R**	**R**	-	-	GTG-CTG	Val 419 Leu
								-	-	GTG-TTG	Val 646 Leu
KPA56	Non-MDR	S	S	S	S	S	**R**	-	-	-	-
KPA57	Non-MDR	**R**	**R**	S	S	**R**	**R**	-	-	-	-
KPA58	Non-MDR	S	**R**	S	S	S	**R**	-	-	-	-
KPA59	Non-MDR	S	**R**	S	S	I	**R**	-	-	GTG-TTG	Val 646 Leu
KPA6	MDR	**R**	**R**	**R**	**R**	**R**	**R**	ACC-ATC	Thr 83 Ile	TCG-TTG	Ser 87 Leu
								-	-	CCA-ACA	Pro 752 Thr
KPA60	MDR	**R**	I	**R**	I	**R**	**R**	-	-	-	-
KPA61	Non-MDR	I	I	S	S	I	I	-	-	-	-
KPA62	Non-MDR	S	S	S	S	S	**R**	-	-	-	-
KPA63	Non-MDR	I	**R**	S	S	S	**R**	-	-	-	-
KPA64	Non-MDR	**R**	**R**	S	I	S	**R**	-	-	-	-
KPA65	Non-MDR	I	I	S	S	I	I	-	-	GTG-CTG	Val 419 Leu
								-	-	GTG-TTG	Val 646 Leu
KPA66	Non-MDR	I	I	S	S	I	I	-	-	-	-
KPA67	Non-MDR	I	I	**R**	S	I	I	-	-	-	-
KPA68	MDR	**R**	**R**	**R**	S	**R**	**R**	-	-	-	-
KPA69	Non-MDR	I	I	I	I	I	I	-	-	-	-
KPA7	MDR	**R**	I	**R**	**R**	**R**	**R**	ACC-ATC	Thr 83 Ile	TCG-TTG	Ser 87 Leu
								-	-	ACC-TCC	Thr 556 Ser
KPA70	Non-MDR	S	S	S	S	S	**R**	-	-	-	-
KPA72	Non-MDR	S	S	S	S	S	**R**	-	-	-	-
KPA73	Non-MDR	**R**	**R**	S	S	**R**	**R**	-	-	-	-
KPA74	MDR	**R**	**R**	I	S	**R**	**R**	ACC-ATC	Thr 83 Ile	TCG-TTG	Ser 87 Leu
KPA8	MDR	**R**	**R**	**R**	**R**	I	**R**	ACC-ATC	Thr 83 Ile	TCG-TTG	Ser 87 Leu
								-	-	ACC-TCC	Thr 556 Ser
KPA83	Non-MDR	S	**R**	S	S	S	**R**	-	-	-	-
KPA119	Non-MDR	S	S	S	S	S	**R**	-	-	-	-
KPA120	Non-MDR	S	**R**	S	S	S	**R**	-	-	-	-
KPA122	Non-MDR	S	**R**	S	S	S	**R**	-	-	-	-
KPA124	Non-MDR	S	**R**	S	S	I	**R**	-	-	-	-
KPA134	Non-MDR	S	S	S	S	I	**R**	-	-	-	-
KPA140	Non-MDR	S	**R**	S	S	I	**R**	-	-	-	-

**Strains**	**AST**	**Antibacterial Susceptibility Tests**						**QRDR Mutations**			
KPA143	Non-MDR	S	**R**	S	S	S	**R**	-	-	-	-
KPA15	Non-MDR	**R**	**R**	S	S	**R**	**R**	-	-	-	-
KPA151	Non-MDR	S	**R**	S	S	I	**R**	-	-	-	-
KPA159	Non-MDR	S	**R**	S	S	I	**R**	-	-	-	-
KPA16	MDR	**R**	**R**	**R**	**R**	**R**	**R**	ACC-ATC	Thr 83 Ile	TCG-TTG	Ser 87 Leu
								-	-	CCA-ACA	Pro 752 Thr
KPA17	Non-MDR	S	S	S	**R**	I	**R**	-	-	GTG-CTG	Val 419 Leu
								-	-	GTG-TTG	Val 646 Leu
KPA18	Non-MDR	S	S	S	S	I	**R**	-	-	GTG-CTG	Val 419 Leu
								-	-	GTG-TTG	Val 646 Leu
KPA19	Non-MDR	S	S	S	S	I	**R**	-	-	-	-
KPA2	MDR	**R**	**R**	**R**	**R**	**R**	**R**	ACC-ATC	Thr 83 Ile	TCG-TTG	Ser 87 Leu
								-	-	CCA-ACA	Pro 752 Thr
KPA20	Non-MDR	S	S	S	S	S	**R**	-	-	-	-
KPA21	Non-MDR	S	S	S	**R**	I	**R**	-	-	-	His 262 Gln
KPA22	MDR	**R**	**R**	**R**	**R**	I	**R**	ACC-ATC	Thr 83 Ile	TCG-TTG	Ser 87 Leu
								-	-	CCA-ACA	Pro 752 Thr
KPA23	Non-MDR	S	S	S	S	S	**R**	-	-	-	-
KPA24	MDR	**R**	**R**	**R**	**R**	I	**R**	ACC-ATC	Thr 83 Ile	TCG-TTG	Ser 87 Leu
								-	-	CCA-ACA	Pro 752 Thr
KPA3	MDR	**R**	I	S	**R**	**R**	**R**	ACC-ATC	Thr 83 Ile	TCG-TTG	Ser 87 Leu
								-	-	CCA-ACA	Pro 752 Thr
KPA4	MDR	**R**	I	**R**	**R**	**R**	**R**	ACC-ATC	Thr 83 Ile	TCG-TTG	Ser 87 Leu
								-	-	CCA-ACA	Pro 752 Thr
KPA44	Non-MDR	S	I	S	S	S	**R**	-	-	-	-
KPA45	Non-MDR	**R**	**R**	I	S	**R**	**R**	-	-	-	-
KPA46	MDR	**R**	**R**	S	**R**	**R**	**R**	-	-	-	-
KPA47	Non-MDR	S	**R**	S	**R**	S	**R**	-	-	-	-
KPA49	Non-MDR	**R**	**R**	S	S	**R**	**R**	-	-	-	-
KPA5	MDR	**R**	**R**	**R**	**R**	I	**R**	ACC-ATC	Thr 83 Ile	TCG-TTG	Ser 87 Leu
								-	-	CCA-ACA	Ser 87 Leu
KPA50	MDR	**R**	**R**	**R**	S	**R**	**R**	ACC-ATC	Thr 83 Ile	TCG-TTG	Pro 752 Thr
								GAC-TAC	Asp 652 Tyr	-	-
KPA51	Non-MDR	**R**	**R**	**R**	S	S	**R**	-	-	-	-
KPA52	Non-MDR	**R**	**R**	S	S	**R**	**R**	-	-	-	-
KPA53	MDR	**R**	**R**	**R**	**R**	I	**R**	ACC-ATC	Thr 83 Ile	TCG-TTG	Ser 87 Leu
								-	-	CCA-ACA	Pro 752 Thr
KPA54	MDR	**R**	**R**	S	**R**	**R**	**R**	ACC-ATC	Thr 83 Ile	GTG-CTG	Val 419 Leu
								-	-	GTG-TTG	Val 646 Leu

Criteria for defining MDR and XDR in *Pseudomonas aeruginosa* as categorized by Magiorakos et al., 2012. AST:

Strains	AST	Antibacterial Susceptibility Tests	QRDR Mutations
Antibiotics susceptibility tests, MDR: Multidrug resistance, S: Susceptible, I: Intermediate, R: Resistant, TCC: Ticarcillin/Clavulinic acid, PIP: Piperacillin, FEP: Cefepime, Meropenem, LXV: Levofloxacin, and TIG: Tigecycline. QRDR: Quinolone Resistance Determining Region, NC: Nucleotide change, AC: Amino acid change, A: Adenine, T: Thymine, C: Cytosine, and G: Guanine. Thr: Threonine, Ile: Isoleucine, Val: Valine, Leu: Leucine, Pro: Proline, Asp: Aspartate, Tyr: Tyrosine, His: Histidine, and Gln: Glutamine.			

Supplementary Table S7: List of resistance genes identified and associated functions

Gene	Product/Function
ArmR	a 53-amino-acid antirepressor allosterically inhibits MexR dimer-DNA binding by occupying a hydrophobic binding cavity within the center of the MexR dimer.
CrpP	confers resistance to ciprofloxacin by antibiotic inactivation through phosphorylation.
EreA2	an integron-encoded erythromycin esterase that hydrolyses the drug's lactone ring. EreA2 is found in Providencia stuartii
MexA	membrane fusion protein of the MexAB-OprM multidrug efflux complex
MexB	inner membrane multidrug exporter of the efflux complex MexAB-OprM.
MexC	membrane fusion protein of the MexCD-OprJ multidrug efflux complex.
MexD	multidrug inner membrane transporter of the MexCD-OprJ complex.
MexE	membrane fusion protein of the MexEF-OprN multidrug efflux complex.
MexF	multidrug inner membrane transporter of the MexEF-OprN complex.
MexG	membrane protein required for MexGHI-OpmD efflux activity.
MexH	membrane fusion protein of the efflux complex MexGHI-OpmD.
MexI	inner membrane transporter of the efflux complex MexGHI-OpmD
MexJ	membrane fusion protein of the MexJK multidrug efflux protein.
MexK	inner membrane resistance-nodulation-cell division (RND) transporter in the MexJK multidrug efflux protein.
MexL	a specific repressor of mexJK transcription and autoregulates its own expression.
MexM	membrane fusion protein of the MexMN-OprM multidrug efflux complex
MexN	inner membrane transporter of the MexMN-OprM multidrug efflux complex.
MexP	membrane fusion protein of the MexPQ-OpmE multidrug efflux complex
MexQ	inner membrane transporter of the multidrug efflux pump MexPQ-OpmE.
MexV	membrane fusion protein of the MexVW-OprM multidrug efflux complex.
MexW	RND-type membrane protein of the efflux complex MexVW-OprM.
MexX	membrane fusion protein of the MexXY-OprM multidrug efflux complex.
MexY	RND-type membrane protein of the efflux complex MexXY-OprM.
MuxA	membrane fusion protein component of the efflux pump system MuxABC-OpmB in Pseudomonas aeruginosa.
MuxB	one of the two necessary RND components in the Pseudomonas aeruginosa efflux pump system MuxABC-OpmB.
MuxC	one of the two necessary RND components of the MuxABC-OpmB efflux pumps system in Pseudomonas aeruginosa.

Gene	Product/Function
NDM-1	metallo-beta-lactamase isolated from Klebsiella pneumoniae with nearly complete resistance to all beta-lactam antibiotics.
OXA-10	beta-lactamase found in Acinetobacter baumannii and P. aeruginosa. Resistance to cephalosporin
OXA-119	a beta-lactamase found in Enterobacteriaceae conferring resistance to cephalosporin
OXA-4	a beta-lactamase found in Enterobacteriaceae and P. aeruginosa conferring resistance to cephalosporin
OXA-486	Assigned by Lahey's list of beta-lactamases conferring resistance to cephalosporin and penam
OXA-488	Assigned by Lahey's list of beta-lactamases conferring resistance to cephalosporin and penam
OXA-50	beta-lactamase found in Pseudomonas aeruginosa. It confers decreased susceptibility to ampicillin and ticarcillin and interestingly to moxalactam and meropenem in P. aeruginosa but not in E. coli. It also confers resistance to piperacillin-tazobactam and cephalotin.
OpmB	an outer membrane efflux protein in Pseudomonas aeruginosa that shows functional cooperation with MuxABC to form the efflux pump system MuxABC-OpmB.
OpmH	outer membrane efflux protein required for triclosan-specific efflux pump function.
OprJ	outer membrane channel component of the MexCD-OprJ multidrug efflux complex.
OprM	an outer membrane factor protein found in Pseudomonas aeruginosa and Burkholderia vietnamiensis. It is part of the MexAB-OprM MexVW-OprM MexXY-OprM and the AmrAB-OprM complex.
OprN	outer membrane channel component of the MexEF-OprN multidrug efflux complex.
PDC-1	extended-spectrum beta-lactamase (ESBL) found in Pseudomonas aeruginosa conferring resistance to carbapenem, cephalosporin, monobactam
PDC-10	extended-spectrum beta-lactamase (ESBL) found in Pseudomonas aeruginosa conferring resistance to carbapenem, cephalosporin, monobactam
PDC-2	extended-spectrum beta-lactamase (ESBL) found in Pseudomonas aeruginosa conferring resistance to carbapenem, cephalosporin, monobactam
PDC-3	extended-spectrum beta-lactamase (ESBL) found in Pseudomonas aeruginosa conferring resistance to carbapenem, cephalosporin, monobactam
PDC-5	extended-spectrum beta-lactamase (ESBL) found in Pseudomonas aeruginosa conferring resistance to carbapenem, cephalosporin, monobactam
PDC-6	extended-spectrum beta-lactamase (ESBL) found in Pseudomonas aeruginosa conferring resistance to carbapenem, cephalosporin, monobactam
PDC-7	extended-spectrum beta-lactamase (ESBL) found in Pseudomonas aeruginosa conferring resistance to carbapenem, cephalosporin, monobactam
PDC-8	extended-spectrum beta-lactamase (ESBL) found in Pseudomonas aeruginosa conferring resistance to carbapenem, cephalosporin, monobactam
PmpM	multidrug efflux pump belonging to the MATE family of Pseudomonas aeruginosa. PmpM is an H+ drug antiporter and is the first reported case of an H+ coupled efflux pump in the MATE family. PmpM confers resistance to fluoroquinolones fradiomycin benzalkonium chloride chlorhexidine gluconate ethidium bromide tetraphenylphosphonium chloride (TPPCl) and rhodamine 6G.
CpxR	directly involved in activating the expression of RND efflux pump MexAB-OprM in P. aeruginosa. CpxR is required to enhance mexAB-oprM expression and drug resistance in the absence of repressor MexR.
catB7	chromosome-encoded variant of the cat gene found in Pseudomonas aeruginosa for chloramphenicol acetyltransferase
emrE	a small multidrug transporter that functions as a homodimer and that couples the efflux of small polyaromatic cations from the cell with the import of protons down an electrochemical gradient. Confers resistance to tetraphenylphosphonium methyl viologen gentamicin kanamycin and neomycin.
soxR	redox-sensitive transcriptional activator that induces expression of a small regulon that includes the RND efflux pump-encoding operon mexGHI-opmD. SoxR was shown to be activated by pyocyanin.
QnrVC1	integron-mediated quinolone resistance protein found in Vibrio cholerae
TriA	membrane protein that is fused to TriB and both are required for the triclosan efflux pump function of TriABC-OpmH in P. aeruginosa.
TriB	membrane protein that is fused to TriA and both are required for the triclosan efflux pump function of TriABC-OpmH in P. aeruginosa.

Gene	Product/Function
TriC	Resistance nodulation cell division (RND) transporter that is a part of TriABC-OpmH a triclosan-specific efflux protein.
VEB-1b	A beta-lactamase that is found in Pseudomonas aeruginosa confering resistance to cephalosporin and monobactam
VEB-9	Beta-lactamase. From the Lahey list of VEB beta-lactamases.
VIM-6	Beta-lactamase found in Pseudomonas spp. Confer resistance to carbapenem, cephalosporin, cephamycin
ble	Confers resistance to bleomycin and bleomycin-like antibiotics in Enterobacteriaceae
dfrA5	An integron-encoded dihydrofolate reductase found in Vibrio cholerae
dfrB2	An integron-encoded dihydrofolate reductase found in an uncultured bacterium from a wastewater treatment plant
dfrB5	A dihydrofolate reductase and trimethoprim resistance gene identified from an integron in Pseudomonas aeruginosa
floR	Chromosome-encoded chloramphenicol exporter that is found in Bordetella bronchiseptica Escherichia coli Klebsiella pneumoniae Salmonella enterica subsp. enterica serovar Typhimurium str. DT104 and Vibrio cholerae
fosA	An enzyme that confers resistance to fosfomycin in Serratia marcescens by breaking the epoxide ring of the molecule.
opmD	Outer membrane channel protein of the efflux complex MexGHI-OpmD.
opmE	Outer membrane factor protein that is part of the multidrug efflux pump MexPQ-OpmE.
qacH	A subunit of the qac multidrug efflux pump in Vibrio cholerae, especially for fluoroquinolone
sul1	Tulfonamide resistant dihydropteroate synthase of Gram-negative bacteria. It is linked to other resistance genes of class 1 integrons.
tet(A)	Tetracycline efflux pump found in many species of Gram-negative bacteria.
tet(G)	A tetracycline efflux protein found in Gram-negative bacteria.
AAC(3)-Id	An aminoglycoside acetyltransferase encoded by genomic islands and integrons in S. enterica P. mirabilis and Vibrio fluvialis
AAC(6')-Ib7	A plasmid-encoded aminoglycoside acetyltransferase in E. cloacae and C. freundii
AAC(6')-Ib9	An integron-encoded aminoglycoside acetyltransferase in P. aeruginosa
AAC(6')-Il	An aminoglycoside acetyltransferase encoded by plasmids and integrons in Enterobacter cloaecae and Klebsiella aerogenes
ANT(2")-Ia	An integron-encoded nucleotidylylation of 2-deoxystreptamine aminoglycosides at the hydroxyl group at position 2" in P. aeruginosa
ANT(3")-IIa	A aminoglycoside nucleotidyltransferase identified in Acinetobacter spp. via horizontal gene transfer mechanisms.
ANT(4')-IIb	Transposon-encoded aminoglycoside nucleotidyltransferase in P. aeruginosa
APH(3")-Ib	Aminoglycoside phosphotransferase encoded by plasmids transposons integrative conjugative elements and chromosomes in Enterobacteriaceae and Pseudomonas spp.
APH(3')-IIb	A chromosomal-encoded aminoglycoside phosphotransferase in P. aeruginosa
APH(3')-VI	An aminoglycoside phosphoryltransferase that acts on the 3-OH of target of aminoglycosides.
APH(6)-Id	Aminoglycoside phosphotransferase encoded by plasmids integrative conjugative elements and chromosomal genomic islands in K. pneumoniae Salmonella spp. E. coli Shigella flexneri Providencia alcalifaciens Pseudomonas spp. V. cholerae Edwardsiella tarda Pasteurella multocida and Aeromonas bestiarum
arnA	Modifies lipid A with 4-amino-4-deoxy-L-arabinose (Ara4N) which allows gram-negative bacteria to resist the antimicrobial activity of cationic antimicrobial peptides and antibiotics such as polymyxin. arnA is found in E. coli and P. aeruginosa.
arr-3	Ribosyltransferase found in Vibrio fluvialis
basS	Histidine protein kinase sensor Lipid A modification gene; part of a two-component system involved in polymyxin resistance that senses high extracellular Fe(2+)
bcr-1	Transmembrane protein which expels bicyclomycin from the cell leading to bicyclomycin resistance. Identified in Pseudomonas aeruginosa strains responsible for outbreaks in Brazil often appearing with blaSPM-1, another

Gene	Product/Function
	bicyclomycin resistance gene
cmlA5	Transposon-encoded chloramphenicol exporter that is found in Escherichia coli

Supplementary Table S8: List of virulence genes identified in the study

Genes	Product/Function
alg44	alginate biosynthesis protein Alg8
alg8	alginate-c5-mannuronan-epimerase AlgG
algA	phosphomannose isomerase / guanosine 5'-diphospho-D-mannose pyrophosphorylase
algB	two-component response regulator AlgB
algC	phosphomannomutase AlgC
algD	GDP-mannose 6-dehydrogenase AlgD
algE	alginate biosynthetic protein AlgK precursor
algF	alginate o-acetyltransferase AlgF
algG	outer membrane protein AlgE
algI	alginate o-acetyltransferase AlgI
algJ	alginate o-acetyltransferase AlgJ
algK	alginate biosynthesis protein Alg44
algL	poly(beta-d-mannuronate) lyase precursor AlgL
algP/algR3	alginate regulatory protein AlgP
algQ	Alginate regulatory protein AlgQ
algR	alginate biosynthesis regulatory protein AlgR
algU	alginate biosynthesis protein AlgZ/FimS
algW	AlgW protein. Alginate regulation
algX	alginate biosynthesis protein AlgX
algZ	sigma factor AlgU
aprA	alkaline metalloproteinase precursor. Alkaline protease
chpA	still frameshift probable component of chemotactic signal transduction system [Type IV pili]
chpB	probable methylesterase [Type IV pili]
chpC	probable chemotaxis protein [Type IV pili]
chpD	probable transcriptional regulator [Type IV pili]

Genes	Product/Function
chpE	probable chemotaxis protein [Type IV pili]
clpV1	type VI secretion system AAA+ family ATPase
dotU1	type VI secretion system protein DotU
exoS	type III secretion system effector ExoS ADP ribosyltransferase activity and GTPase-activating protein activity
exoT	type III secretion system effector ExoT ADP ribosyltransferase activity and GTPase-activating protein activity
exoU	type III secretion system effector ExoU phospholipase A2 activity [ExoU]
exoY	type III secretion system effector ExoY adenylate cyclase
exsA	type III secretion system regulatory protein ExsA
exsB	type III secretion system piolitin ExsB
exsC	type III secretion system regulatory protein ExsC
exsD	type III secretion system regulatory protein ExsD
exsE	type III secretion system regulatory protein ExsE
fha1	type VI secretion system forkhead-associated protein Fha1
fimT	type 4 fimbrial biogenesis protein FimT
fimU	type 4 fimbrial biogenesis protein FimU
fimV	putative Type IV pili related protein
fleI/flag	flagellar protein FlaG [Deoxyhexose linking sugar 209 Da capping structure (AI138)]
fleN	flagellar synthesis regulator FleN
fleP	flagellar protein FliT [Deoxyhexose linking sugar 209 Da capping structure (AI138)]
fleQ	transcriptional regulator FleQ
fleR	two-component response regulator
fleS	two-component sensor [Deoxyhexose linking sugar 209 Da capping structure (AI138)]
flgA	flagellar basal body P-ring biosynthesis protein FlgA [Deoxyhexose linking sugar 209 Da capping structure (AI138)]
flgB	flagellar basal body rod protein FlgB [Deoxyhexose linking sugar 209 Da capping structure (AI138)]
flgC	flagellar basal-body rod protein FlgC
flgD	flagellar basal-body rod modification protein FlgD
flgE	flagellar hook protein FlgE
flgF	flagellar basal-body rod protein FlgF
flgG	flagellar basal-body rod protein FlgG
flgH	flagellar L-ring protein precursor FlgH
flgI	flagellar P-ring protein precursor FlgI
flgJ	flagellar rod assembly protein/muramidase FlgJ
flgK	flagellar hook-associated protein 1 FlgK
flgL	flagellar hook-associated protein 3 FlgL
flgM	negative regulator of flagellin synthesis [Deoxyhexose linking sugar 209 Da capping structure (AI138)]
flgN	flagella synthesis protein FlgN [Deoxyhexose linking sugar 209 Da capping structure
flhA	flagellar biosynthesis protein FlhA

Genes	Product/Function
flhB	flagellar biosynthetic protein FlhB
flhF	flagellar biosynthesis protein FlhF
fliA	flagellar biosynthesis sigma factor FliA [Deoxyhexose linking sugar 209 Da capping structure (AI138)]
fliC	B-type flagellin
fliD	flagellar capping protein FliD
fliE	flagellar hook-basal body complex protein FliE
fliF	flagellar M-ring protein FliF
fliG	flagellar motor switch protein G
fliH	flagellar assembly protein H
fliI	flagellum-specific ATP synthase FliI
fliJ	flagellar protein FliJ
fliK	flagellar hook-length control protein FliK [Deoxyhexose linking sugar 209 Da capping structure (AI138)]
fliL	flagellar basal body protein FliL [Deoxyhexose linking sugar 209 Da capping structure (AI138)]
fliM	flagellar motor switch protein FliM
fliN	flagellar motor switch protein FliN
fliO	flagellar protein FliO
fliP	flagellar biosynthetic protein FliP
fliQ	flagellar biosynthetic protein FliQ
fliR	flagellar biosynthetic protein FliR
fliS	flagellar protein FliS [Deoxyhexose linking sugar 209 Da capping structure (AI138)]
fptA	Fe(III)-pyochelin receptor precursor [Pyochelin (VF0095)]
fpvA	ferripyoverdine receptor FpvA
hcp1	type VI secretion system substrate Hcp1
hsiA1	type VI secretion system hcp secretion island protein HsiA1
hsiB1/vipA	type VI secretion system tubule-forming protein VipA
hsiC1/vipB	type VI secretion system tubule-forming protein VipB
hsiE1	type VI secretion system hcp secretion island protein HsiE1 interacting with HsiB1 to form a novel subcomplex of the T6SS
hsiF1	type VI secretion system hcp secretion island protein HsiF1 a gp25-like protein but not exhibit lysozyme activity
hsiG1	type VI secretion system hcp secretion island protein HsiG1
hsiH1	type VI secretion system hcp secretion island protein HsiH1
hsiJ1	type VI secretion system hcp secretion island protein HsiJ1
icmF1/tssM1	type VI secretion system protein IcmF1
lasA	LasA protease precursor
lasB	elastase LasB
lasI	autoinducer synthesis protein LasI [Quorum sensing (VF0093)]
lip1	lipoprotein
mbtH-like	MbtH-like protein from the pyoverdine cluster

Genes	Product/Function
motA	flagellar motor protein [Deoxyhexose linking sugar 209 Da capping structure]
motB	flagellar motor protein [Deoxyhexose linking sugar 209 Da capping structure]
motC	flagellar motor protein [Deoxyhexose linking sugar 209 Da capping structure
motD	flagellar motor protein [Deoxyhexose linking sugar 209 Da capping structure
motY	probable outer membrane protein precursor [Deoxyhexose linking sugar 209 Da capping structure
mucA	alkaline metalloproteinase precursor
mucB	anti-sigma factor MucA inhibitor of alg gene expression
mucC	negative regulator for alginate biosynthesis MucB
mucD	serine protease MucD precursor
mucE	small envelope protein MucE [Alginate regulation (CVF523)]
mucP	metalloprotease protease [Alginate regulation (CVF523)]
pchA	salicylate biosynthesis isochorismate synthase PchA [Pyochelin (VF0095)]
pchB	salicylate biosynthesis protein PchB [Pyochelin (VF0095)]
pchC	pyochelin biosynthetic protein PchC [Pyochelin (VF0095)]
pchD	pyochelin biosynthesis protein PchD [Pyochelin (VF0095)]
pchE	dihydroaeruginoic acid synthetase PchE [Pyochelin (VF0095)]
pchF	pyochelin synthetase PchF [Pyochelin (VF0095)]
pchG	pyochelin biosynthetic protein PchG [Pyochelin (VF0095)]
pchH	ABC transporter ATP-binding protein [Pyochelin (VF0095)]
pchI	ABC transporter ATP-binding protein [Pyochelin (VF0095)]
pchR	transcriptional regulator PchR [Pyochelin (VF0095)]
pcr1	type III secretion system protein Pcr1 [TTSS (VF0083)]
pcr2	type III secretion system protein Pcr2 [TTSS (VF0083)]
pcr3	type III secretion system protein Pcr3 [TTSS (VF0083)]
pcr4	type III secretion system protein Pcr4 [TTSS (VF0083)]
pcrD	type III secretion system protein PcrD [TTSS (VF0083)]
pcrG	type III secretion system cytoplasmic regulator PcrG [TTSS (VF0083)]
pcrH	type III secretion system regulatory protein PcrH [TTSS (VF0083)]
pcrR	type III secretion system regulatory protein PcrR [TTSS (VF0083)]
pcrV	type III secretion system hydrophilic translocator needle tip protein PcrV [TTSS]
phzA1	phenazine biosynthesis protein PhzA [Phenazines biosynthesis (CVF536)]
phzB1	phenazine biosynthesis protein PhzB [Phenazines biosynthesis (CVF536)]
phzC1	phenazine biosynthesis protein PhzC [Phenazines biosynthesis (CVF536)]
phzD1	phenazine biosynthesis protein PhzD isochorismatase [Phenazines biosynthesis]
phzE1	phenazine biosynthesis protein PhzE [Phenazines biosynthesis (CVF536)]
phzF1	phenazine biosynthesis protein PhzF isomerase [Phenazines biosynthesis (CVF536)]
phzG1	phenazine biosynthesis protein PhzG pyridoxamine 5'-phosphate oxidase [Phenazines biosynthesis (CVF536)]
phzH	phenazine-modifying enzyme [Phenazines biosynthesis (CVF536)]
phzM	phenazine-specific methyltransferase PhzM (adenosylmethionine dependent methyltransferase) [Pyocyanin (VF0100)]

Genes	Product/Function
phzS	flavin dependent hydroxylase PhzS [Pyocyanin (VF0100)]
pilA	type 4 fimbrial precursor PilA [Type IV pili (VF0082)]
pilB	type 4 fimbrial biogenesis protein PilB [Type IV pili (VF0082)]
pilC	still frameshift type 4 fimbrial biogenesis protein PilC [Type IV pili (VF0082)]
pilE	type 4 fimbrial biogenesis protein PilE [Type IV pili (VF0082)]
pilF	type 4 fimbrial biogenesis protein PilF [Type IV pili (VF0082)]
pilG	twitching motility protein PilG [Type IV pili (VF0082)]
pilH	twitching motility protein PilH [Type IV pili (VF0082)]
pilI	twitching motility protein PilI [Type IV pili (VF0082)]
pilJ	twitching motility protein PilJ [Type IV pili (VF0082)]
pilK	methyltransferase PilK [Type IV pili (VF0082)]
pilM	type IV pilus inner membrane platform protein PilM [Type IV pili (VF0082)]
pilN	type IV pilus inner membrane platform protein PilN [Type IV pili (VF0082)]
pilO	type IV pilus inner membrane platform protein PilO [Type IV pili (VF0082)]
pilP	type IV pilus biogenesis protein PilP [Type IV pili (VF0082)]
pilQ	type 4 fimbrial biogenesis protein PilQ [Type IV pili (VF0082)]
pilR	two-component response regulator PilR [Type IV pili (VF0082)]
pilS	two-component sensor PilS [Type IV pili (VF0082)]
pilT	twitching motility protein PilT [Type IV pili (VF0082)]
pilU	twitching motility protein PilU [Type IV pili (VF0082)]
pilV	type IV pilus biogenesis protein PilV [Type IV pili (VF0082)]
pilW	type IV fimbrial biogenesis protein PilW [Type IV pili (VF0082)]
pilX	type 4 fimbrial biogenesis protein PilX [Type IV pili (VF0082)]
pilY1	type 4 fimbrial biogenesis protein PilY1 [Type IV pili (VF0082)]
pilY2	type 4 fimbrial biogenesis protein PilY2 [Type IV pili (VF0082)]
plcH	hemolytic phospholipase C precursor [PLC (VF0092)]
popB	type III secretion system hydrophobic translocator pore protein PopB [TTSS]
popD	type III secretion system hydrophobic translocator pore protein PopD [TTSS]
popN	type III secretion system outer membrane protein PopN [TTSS]
ppkA	serine/threonine protein kinase PpkA [HSI-I (VF0334)]
pppA	Pseudomonas protein phosphatase PppA [HSI-I (VF0334)]
pscB	type III secretion system protein PscB [TTSS (VF0083)]
pscC	type III secretion system secretin PscC [TTSS (VF0083)]
pscD	type III secretion system basal body protein PscD [TTSS (VF0083)]
pscE	type III secretion system cochaperone PscE for PscG [TTSS (VF0083)
pscF	type III secretion system needle filament protein PscF [TTSS (VF0083)]
pscG	type III secretion system chaperone PscG for PscF [TTSS (VF0083)]
pscH	type III secretion system protein PscH [TTSS (VF0083)]
pscI	type III secretion system inner rod protein PscI [TTSS (VF0083)]
pscJ	type III secretion system inner MS ring protein [TTSS (VF0083)]

Genes	Product/Function
pscK	type III secretion system protein PscK [TTSS (VF0083)]
pscL	type III secretion systemt protein PscL [TTSS (VF0083)]
pscM	type III secretion system ATPase PscM [TTSS (VF0083)]
pscN	type III secretion system ATPase PscN [TTSS (VF0083)]
pscO	type III secretion system protein PscO [TTSS (VF0083)]
pscP	type III secretion system protein PscP [TTSS (VF0083)]
pscQ	type III secretion system protein PscQ [TTSS (VF0083)]
pscR	type III secretion system protein PscR [TTSS (VF0083)]
pscS	type III secretion system protein PscS [TTSS (VF0083)]
pscT	type III secretion system protein PscT [TTSS (VF0083)]
pscU	type III secretion system protein PscU [TTSS (VF0083)]
ptxR	transcriptional regulator PtxR [pyoverdine (IA001)]
pvcA	paerucumarin biosynthesis protein PvcA [pyoverdine (IA001)]
pvcB	paerucumarin biosynthesis protein PvcB [pyoverdine (IA001)]
pvcC	paerucumarin biosynthesis protein PvcC [pyoverdine (IA001)]
pvcD	paerucumarin biosynthesis protein PvcD [pyoverdine (IA001)]
pvdA	L-ornithine N5-oxygenase PvdA [Pyoverdine (VF0094)]
pvdD	pyoverdine synthetase D [Pyoverdine (VF0094)]
pvdE	pyoverdine biosynthesis protein PvdE [Pyoverdine (VF0094)]
pvdF	pyoverdine synthetase F [pyoverdine (IA001)]
pvdG	pyoverdine biosynthesis protein PvdG [pyoverdine (IA001)]
pvdH	diaminobutyrate-2-oxoglutarate aminotransferase PvdH [pyoverdine (IA001)]
pvdI	peptide synthase [pyoverdine (IA001)]
pvdJ	pyoverdine biosynthesis protein PvdJ [pyoverdine (IA001)]
pvdL	peptide synthase PvdL [pyoverdine (IA001)]
pvdM	dipeptidase precursor [pyoverdine (IA001)]
pvdN	pyoverdine biosynthesis protein PvdN [pyoverdine (IA001)]
pvdO	pyoverdine biosynthesis protein PvdO [pyoverdine (IA001)]
pvdP	tyrosinase required for pyoverdine maturation [pyoverdine (IA001)]
pvdQ	3-oxo-C12-homoserine lactone acylase PvdQ [pyoverdine (IA001)]
pvdS	extracytoplasmic-function sigma-70 factor [Pyoverdine (VF0094)]
rhlA	rhamnosyltransferase chain A [Rhamnolipid (VF0089)]
rhlB	rhamnosyltransferase chain B [Rhamnolipid (VF0089)]
rhlC	rhamnosyltransferase 2 [Rhamnolipid biosynthesis (CVF524)]
rhlI	autoinducer synthesis protein RhlL [Quorum sensing (VF0093)]
tagF/pppB	Pseudomonas protein phosphatase PppB [HSI-I (VF0334)]
tagQ	type VI secretiona ssociated protein TagQ outer membrane lipoprotein [HSI-1 (Hcp-secretion island 1) (SS178)]
tagR	type IV secretion associated protein TagR positively regulates PpkA [HSI-I]
tagS	type IV secretion associated protein TagS forming a stable inner membrane complex with TagT [HSI-I (VF0334)]

Genes	Product/Function
tagT	type six secretion associated protein TagT ATP-binding component of ABC transporter [HSI-I (VF0334)]
toxA	exotoxin A precursor [ExoA (VF0086)]
tse1	type VI secretion system effector Tse1 peptidoglycanhydrolase [HSI-1 (Hcp-secretion island 1) (SS178)]
tse2	type VI secretion system effector Tse2 [HSI-1 (Hcp-secretion island 1) (SS178)]
tse3	type VI secretion system effector Tse3 glycoside hydrolase [HSI-1 (Hcp-secretion island 1) (SS178)]
vgrG1a	type VI secretion system substrate VgrG1 [HSI-I (VF0334)]
vgrG1b	type VI secretion system substrate VgrG1b [HSI-1 (Hcp-secretion island 1) (SS178)]
waaA	lipopolysaccharide core biosynthesis protein WaaP [LPS (VF0085)]
waaC	3-deoxy-D-manno-octulosonic-acid (KDO) transferase [LPS (VF0085)]
waaF	heptosyltransferase I [LPS (VF0085)]
waaG	B-band O-antigen polymerase [LPS (VF0085)]
waaP	UDP-glucose:(heptosyl) LPS alpha 13-glucosyltransferase WaaG [LPS (VF0085)]
wzy	O-antigen chain length regulator [LPS (VF0085)]
wzz	positive regulator for alginate biosynthesis MucC [LPS (VF0085)]
xcpA/pilD	type 4 prepilin peptidase PilD [Type IV pili (VF0082)]
xcpP	secretion protein XcpP [xcp secretion system (VF0084)]
xcpQ	general secretion pathway protein D [xcp secretion system (VF0084)]
xcpR	general secretion pathway protein E [xcp secretion system (VF0084)]
xcpS	general secretion pathway protein F [xcp secretion system (VF0084)]
xcpT	general secretion pathway protein G [xcp secretion system (VF0084)]
xcpU	general secretion pathway protein H [xcp secretion system (VF0084)]
xcpV	general secretion pathway protein I [xcp secretion system (VF0084)]
xcpW	general secretion pathway protein J [xcp secretion system (VF0084)]
xcpX	general secretion pathway protein K [xcp secretion system (VF0084)]
xcpY	general secretion pathway protein L [xcp secretion system (VF0084)]
xcpZ	general secretion pathway protein M [xcp secretion system (VF0084)]
